# Rv2346c enhances mycobacterial survival within macrophages by inhibiting TNF-α and IL-6 production via the p38/miRNA/NF-κB pathway

**DOI:** 10.1038/s41426-018-0162-6

**Published:** 2018-09-19

**Authors:** Jing Yao, Xingran Du, Sixia Chen, Yan Shao, Kaili Deng, Mingzi Jiang, Jingning Liu, Ziyan Shen, Xiaolin Chen, Ganzhu Feng

**Affiliations:** 1 Department of Respiratory Medicine, the Second Affiliated Hospital of Nanjing Medical University, Nanjing, Jiangsu 210011 China; 2 Department of Infectious Diseases, the Second Affiliated Hospital of Nanjing Medical University, Nanjing, Jiangsu 210011 China; 3 Jiangsu Provincial Center for Disease Control and Prevention, Nanjing, Jiangsu 210009 China; 4 Department of Respiratory Medicine, the First People’s Hospital of Kunshan, Kunshan, Jiangsu 215300 China; 5 Department of Respiratory Medicine, Sir Run Run Hospital, Nanjing, Jiangsu 211100 China

## Abstract

The intracellular survival of *Mycobacterium tuberculosis* (*Mtb*) has a central role in the pathogenesis of tuberculosis. *Mtb* Rv2346c is a member of 6-kDa early secreted antigenic target family of proteins, which are known to inhibit the host immune responses to promote bacillary persistence in macrophages. However, the mechanism through which Rv2346c participates in *Mtb* pathogenesis is unclear. In the present study, recombinant Rv2346c protein was synthesized and used to treat Bacillus Calmette–Guérin (BCG)-infected macrophages. The results showed that Rv2346c inhibited the proliferation of BCG-infected macrophages and enhanced the survival of BCG in macrophages. Tumor necrosis factor-α (TNF-α) and interleukin (IL)-6 were upregulated during BCG infection but downregulated by Rv2346c. Additional experiments showed that nuclear transcription factor-κB (NF-κB) in BCG-infected macrophages induced the production of TNF-α and IL-6. In addition, miR-155 and miR-99b had a suppressive effect on NF-κB, and the expression of these miRNAs was promoted by p38. Furthermore, Rv2346c was shown to decrease the activation of NF-κB, whereas it enhanced the phosphorylation of p38 and the expression of miR-155 and miR-99b. The function of Rv2346c was also verified in *Mtb*-infected mice. The results showed that Rv2346c increased the observed bacterial load and lung injury and downregulated TNF-α and IL-6 in vivo. Overall, our results reveal that Rv2346c enhances mycobacterial survival in macrophages via inhibiting the production of TNF-α and IL-6 in a p38/miRNA/NF-κB pathway-dependent manner, suggesting that Rv2346c acts as a crucial virulence factor in *Mtb* infection and has potential use as a target for anti-tuberculosis therapy.

## Introduction

Tuberculosis (TB) remains a major public health problem worldwide, particularly in developing countries, and is caused by *Mycobacterium tuberculosis* (*Mtb*) infections. Approximately one-third of the world’s population has been infected with *Mtb*^1^. Bacillus Calmette–Guérin (BCG), a live-attenuated *Mycobacterium*, is the most widely used human vaccine against TB for controlling this common infection^2^. However, BCG is reported to partially protect against TB meningitis and disseminated TB in infants but not adequately protect against pulmonary TB, the most prevalent form of disease, in all age groups^3^. Therefore, gaining an understanding of the molecular mechanisms of the pathogenesis of TB is urgently needed to allow for more effective prophylaxis, diagnosis, and therapy.

*Mtb* is able to bind to phagocytic receptors after being inhaled, enter resident alveolar macrophages recruited from the bloodstream, and live in them as an intracellular pathogen. Macrophages act as the first line of immune defense against *Mtb* by clearing the pathogen and functioning as antigen-presenting cells, transforming *Mtb* antigens into immunogenic polypeptides and presenting them to T cells via the major histocompatibility complex to trigger adaptive immunity^4^. In the process of developing cellular immunity against *Mtb*, macrophages produce tumor necrosis factor-α (TNF-α) through stimulation of Toll-like receptor-2 (TLR2)-mediated signaling pathway and translocation of nuclear transcription factor-κB (NF-κB) to the nucleus^4,5^. TNF-α further induces host cell apoptosis to promote clearance of *Mtb*^6^, which is related to the caspase 8-mediated extrinsic cell death pathway, involving apoptosis signal-regulating kinase 1, the mitogen-activated protein kinase (MAPK) p38, and c-Abl^7^. In response to *Mtb* infection, interleukin (IL)-6 is also produced to kill *Mycobacteria*^8^. Furthermore, microRNAs (miRNAs), which are capable of controlling the activity of protein-encoding genes, have been implicated in modulating molecular mechanisms of pathogenesis in *Mtb* infection^9^.

The virulence determinants that promote the intracellular survival of *Mtb* in macrophages is the pivotal issue regarding the pathogenesis of TB. *Mtb* is reported to exploit the 6 kDa early secreted antigenic target (ESAT-6) secretion system (ESX), which is responsible for exporting virulence factors and immunomodulators across the cytomembrane^10^. These ESX substrates are known to inhibit the host immune responses to promote bacillary persistence in macrophages and the 6 kDa early secretory antigenic target (ESAT-6) is one of those proteins secreted by ESX secretion system. *Mtb* exhibits membrane-perforating activity due to the secretion of ESAT-6^11^, which is responsible for the membrane rupture of phagosomes and the subsequent escape of *Mtb* into the cytoplasm^12^. The ESAT-6-encoding gene is located in the RD1 region, which is conserved in virulent *Mycobacterium bovis* and *Mtb* strains, and is deleted from all BCG substrains^13^. It has been shown that *Mtb* Rv2346c (Gene ID: 888956) is a member of the ESAT-6-like family of proteins; thus, it may possess some common functions with ESAT-6. As Rv2346c also displays some differences with other family members, it may function differently in some respects. The overexpression of Rv2346c in *Mtb* has been reported to enhance its survival in human and mouse macrophages by inducing oxidative stress-mediated genomic instability^14^. However, the role of Rv2346c in *Mtb* pathogenesis and host immunity is still unclear. In this study, recombinant Rv2346c protein was produced to evaluate its immunomodulatory effect on BCG-infected macrophages and the related molecular mechanism. The function of Rv2346c was also verified in vivo.

## Results

### Heterologous expression and purification of recombinant Rv2346c

The Rv2346c protein was synthesized and purified for further investigation. The Rv2346c-encoding gene was amplified by PCR, cloned into the plasmid pET-30a( + ), and transformed into *Escherichia coli* BL21 (DE3). The positive clones were confirmed by sequencing, the results of which are shown in Fig S1a. The recombinant protein Rv2346c was expressed in *E. coli* BL21(DE3) and was subsequently purified with Ni-IDA resin.

### Rv2346c inhibits the proliferation of BCG-infected macrophages and enhances the survival of BCG in macrophages

**Fig. 1:**
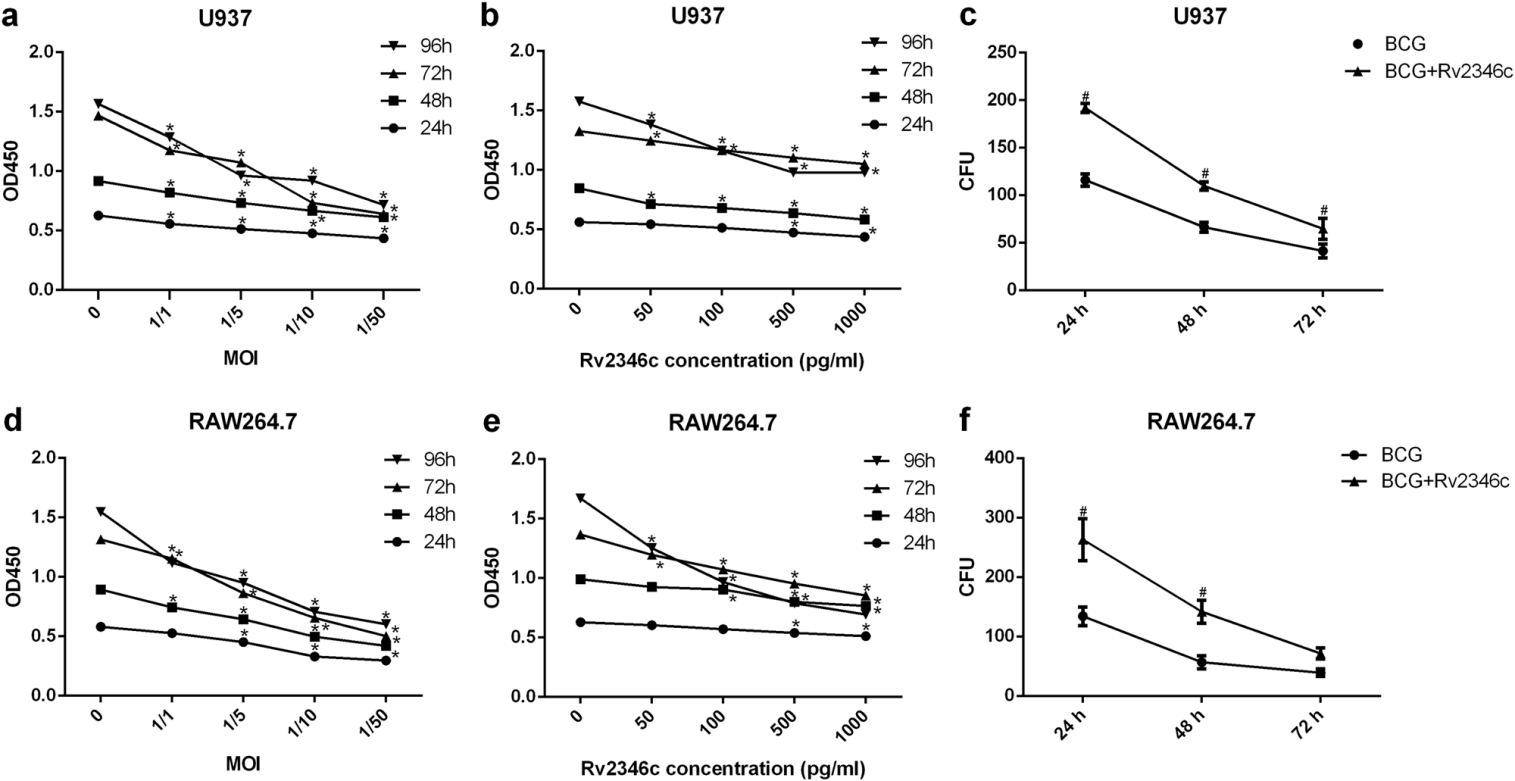
Effect of Rv2346c on the proliferation of BCG-infected macrophages and the survival of BCG in macrophages. U937 and RAW264.7 cells were treated with BCG at the indicated MOI or BCG (MOI 1/5) together with the recombinant Rv2346c protein for 24, 48, 72, or 96 h. A CCK-8 assay was performed to estimate cell proliferation (**a**, **b**, **d**, **e**). Cells were also infected with BCG (MOI 1/5) with or without the recombinant Rv2346c protein (500 pg/ml) treatment for the indicated time, and a CFU assay was performed to assay BCG survival (**c**, **f**). All the presented graphs are representative of three independent experiments. Data are presented as the means ± SD. *P < 0.05 vs. control group. #*P* < 0.05 vs. BCG group of the same infection time

To explore the function of Rv2346c in TB, a cell counting kit-8 (CCK-8) assay was used to estimate its effect on BCG-infected macrophage proliferation. The results demonstrated that BCG infection significantly inhibited U937 and RAW264.7 cell proliferation, and this effect increased with higher multiplicity of infections (MOIs) of BCG and longer incubation times (Fig. 1a, d). When U937 and RAW264.7 cells were treated with BCG and Rv2346c simultaneously, Rv2346c further inhibited cell proliferation in a dose-dependent manner (Fig. 1b, e), whereas Rv2346c alone did not affect cell proliferation (Fig S1b, c). Macrophages have been reported to be capable of phagocytosing and killing *Mtb*. In the present study, the survival of BCG in U937 and RAW264.7 cells was evaluated by a colony-forming unit (CFU) assay. Our results showed that Rv2346c inhibited the germicidal efficacy of U937 and RAW264.7 cells and enhanced the survival of BCG (Fig. 1c, f).

### Rv2346c inhibition of cytokine production is accompanied by the downregulation of p65 and upregulation of p38 phosphorylation in BCG-infected macrophages

**Fig. 2:**
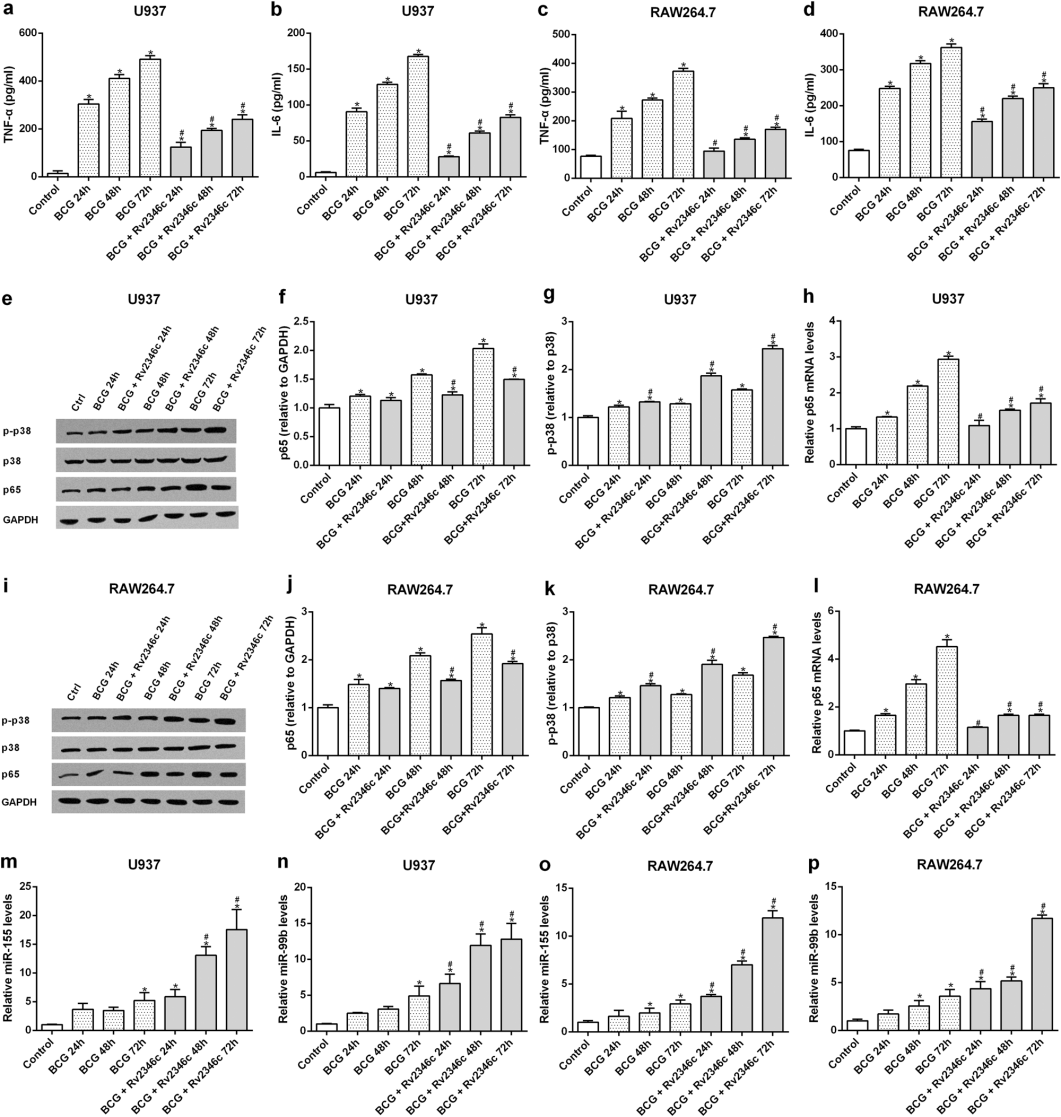
Effect of Rv2346c on cytokine production, p65 expression, p38 phosphorylation, and miR-155 and miR-99b levels. U937 and RAW264.7 cells were infected with BCG (MOI 1/5) and cells in the BCG + Rv2346c groups were also treated with Rv2346c (500 pg/ml). After incubating for 24, 48, or 72 h, cell culture supernatants were collected and examined using ELISA assay (**a**–**d**); total protein was extracted and estimated via western blot (**e**, **f**, **g**, **i**, **j**, **k**); total RNA was extracted and analyzed via qRT-PCR (**h**, **l**, **m**–**p**). GAPDH and p38 were used as loading controls for protein expression analysis. Protein bands were scanned and the intensity was determined. The results are representative of three independent experiments. Data are presented as the means ± SD. *P < 0.05 vs. control group; #*P* < 0.05 vs. BCG group of the same infection time

TNF-α and IL-6 participate in the clearance of *Mtb* by macrophages^15,16^. To determine whether Rv2346c impaired the germicidal efficacy of macrophages by decreasing the production of these cytokines, the TNF-α and IL-6 levels in cell culture supernatants were evaluated by enzyme-linked immunosorbent assay (ELISA). The results showed that TNF-α and IL-6 levels in supernatants were increased after BCG infection in U937 and RAW264.7 cells, whereas these increases were inhibited by the addition of Rv2346c (Fig. 2a–d). These data indicated that Rv2346c enhanced the survival of BCG by inhibiting the production of TNF-α and IL-6.

The production of TNF-α and IL-6 have been reported to be induced by NF-κB activation in macrophages, which can be evaluated by assessing the level of p65, a NF-κB submit^17,18^. Our results also showed that the expression of p65 is in line with the observed p65 phosphorylation, which supports NF-κB activation, during BCG infection, Rv2346c treatment, p65 overexpression, or p65 interference (Figs S2 and S3). In addition, p38 is known to affect proinflammatory cytokines in *Mtb*-infected macrophages and its activity can be estimated by its phosphorylation level^19^. To identify the upstream regulator of these cytokines that is affected by Rv2346c, the protein expression levels of p65 and p-p38 were evaluated by western blotting and the mRNA level of p65 was assessed by quantitative reverse-transcription PCR (qRT-PCR). The results showed that transcription and translation of p65 were upregulated the in BCG-infected macrophages, but this effect was reduced after cells were treated with Rv2346c (Fig. 2e, f, h, i, j, l). In addition, BCG infection upregulated the level of p38 phosphorylation in macrophages, which was further enhanced by treating cells with Rv2346c (Fig. 2e, g, i, k). These data suggested that NF-κB and p38 participated in the Rv2346c-mediated regulation of cytokine expression in BCG-infected macrophages.

### Rv2346c upregulates miR-155 and miR-99b in BCG-infected macrophages

The expressions of numerous proteins are regulated by miRNAs in eukaryotes. To identify miRNAs associated with the process of BCG infection, a microarray analysis was conducted to miRNAs that were differentially expressed between the BCG-infected and BCG + Rv2346c groups. We detected 2006 miRNAs in U937 cells and 1193 miRNAs in RAW264.7 cells. Differentially expressed miRNAs were identified using a screening criterion of |Fold change| ≥ 2 and identified miRNAs are shown in Fig S4. Among these, miRNA-155 and miRNA-99b were significantly upregulated in Rv2346c-treated cells and these miRNAs have been previously observed to be associated with *Mtb* infection. The expression levels of miR-155 and miR-99b in U937 and RAW264.7 cells were evaluated by qRT-PCR. The results showed that BCG infection upregulated the levels of miR-155 and miR-99b in U937 and Raw264.7 cells, which was further enhanced by treatment with Rv2346c (Fig. 2m–p).

### p65 upregulates the levels of TNF-α and IL-6 without affecting p38 phosphorylation and miRNA expression

**Fig. 3:**
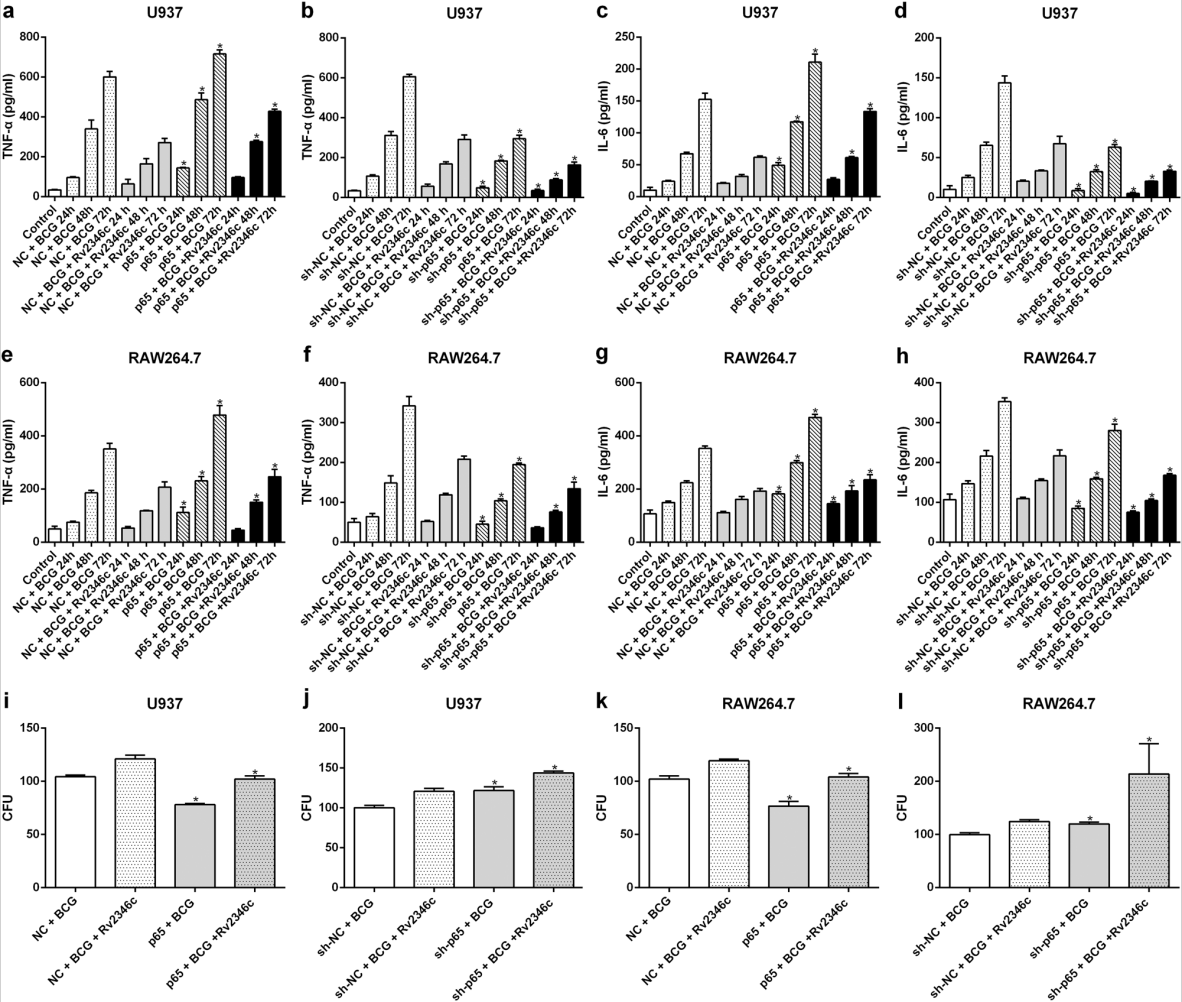
Effect of p65 intervention on the production of TNF-α and IL-6 and the survival of BCG. U937 and RAW264.7 cells were transfected with a control vector (indicated with NC), a p65 overexpression vector (indicated with p65), a nonspecific siRNA (indicated with sh-NC) or a specific siRNA directed against p65 (indicated with sh-p65). After 24 h post transfection, cells were infected with BCG (MOI 1/5) and cells in the BCG + Rv2346c group were also treated with Rv2346c (500 pg/ml). Cell culture supernatants were collected and detected with ELISA after incubating for 24, 48, or 72 h (**a**–**h**). BCG survival was evaluated by a CFU assay after incubating for 24 h (**i**–**l**). The results are representative of three independent experiments. Data are presented as the means ± SD. *P < 0.05 vs. the control vector or the nonspecific siRNA interference group of the same treatment

To verify the effect of p65 on cytokine regulation in *Mtb* infection, p65 was upregulated in U937 and RAW264.7 cells via transfection with pcDNA3.1/p65 cDNA or downregulated through transfection with a specific small interfering RNA (siRNA) directed against p65. The efficacy of the treatments was evaluated by western blotting and qRT-PCR analyses (Fig S5). The result showed that p65 overexpression upregulated TNF-α and IL-6 expression, whereas p65 interference significantly reduced the levels of TNF-α and IL-6 (Fig. 3a–h). In addition, overexpression of p65 enhanced the germicidal effect against BCG, whereas downregulation of p65 suppressed the bactericidal activity of macrophages against BCG, which was in line with its observed regulation of cytokine production (Fig. 3i–l). These results did not indicate the existence of a regulatory effect of p65 on the phosphorylation of p38 (Fig S6) or the expression of miR-155 and miR-99b (Fig S7).

### miR-155 and miR-99b downregulate the expression of p65 and the levels of TNF-α and IL-6 without affecting p38 phosphorylation

**Fig. 4:**
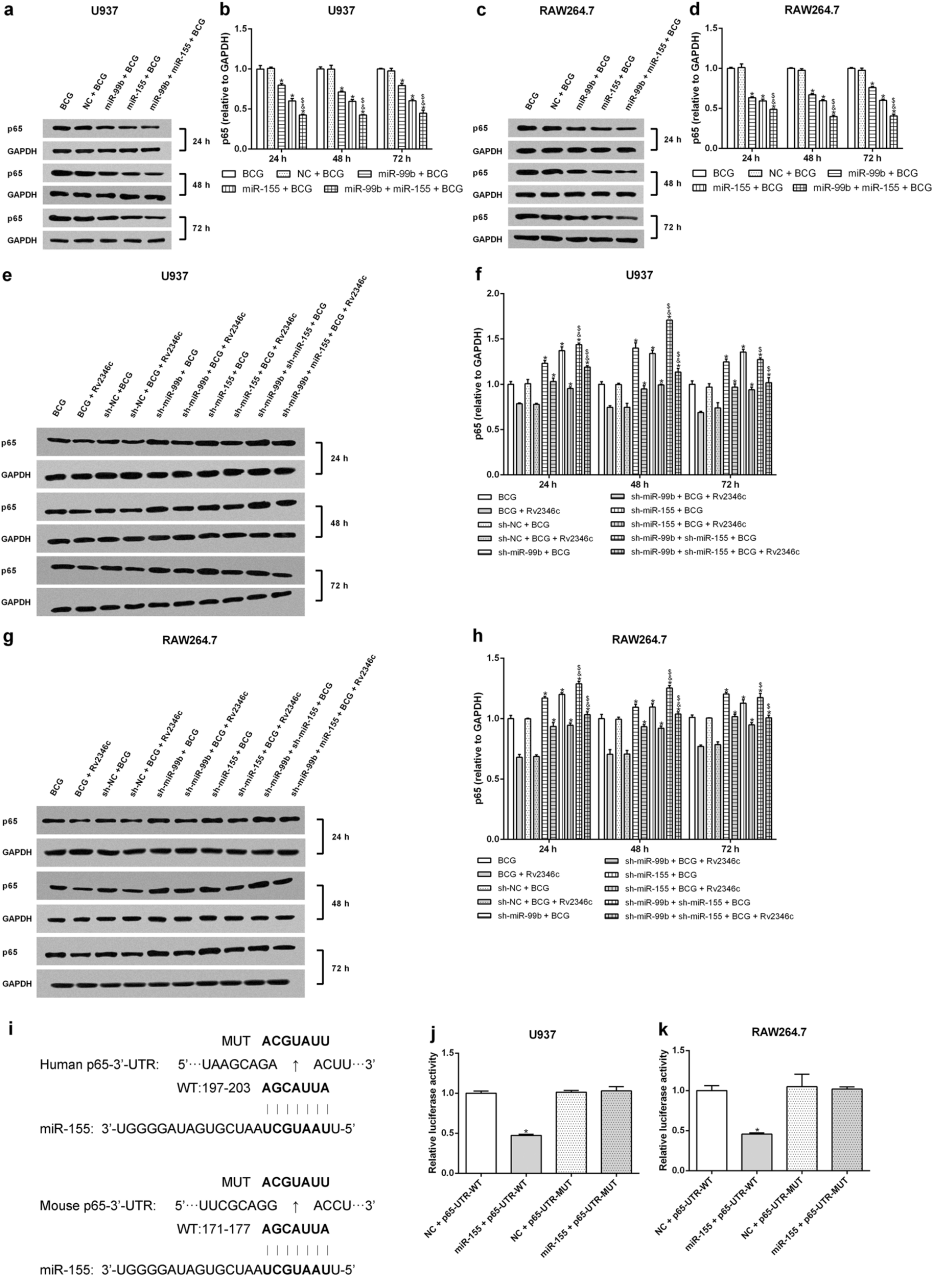
Effect of miR-155 and miR-99b on p65 expression. U937 and RAW264.7 cells were transfected with a negative control miRNA mimic (indicated with NC), miR-155, or miR-99b mimics, both miR-155 and miR-99b mimics, a negative control miRNA inhibitor (indicated with sh-NC), a miR-155 inhibitor (indicated with sh-miR-155), a miR-99b inhibitor (indicated with sh-miR-99b), or both the miR-99b and miR-155 inhibitors. After 24 h post transfection, cells were infected with BCG (MOI 1/5) and cells in the BCG + Rv2346c group were also treated with Rv2346c (500 pg/ml). After incubating for 24, 48, or 72 h, cells were collected to extract total protein. The protein expression was detected via western blotting. The results are representative of three independent experiments. Data are presented as the means ± SD. *P < 0.05 vs. a negative control (NC group or sh-NC group) + BCG group (with or without Rv2346c treatment); &*P* < 0.05 vs. miR-99b or sh-miR-99b + BCG group (with or without Rv2346c treatment); $*P* < 0.05 vs. miR-155 or sh-miR-155 + BCG group (with or without Rv2346c treatment) (**a**–**h**). Sequence alignment of miR-155 and its conserved target site in the P65-UTR is shown (**i**). Luciferase activity was measured in U937 and RAW264.7 cells with a dual-luciferase reporter assay. The cells were co-transfected with a plasmid expressing miR-155 mimic or a control miRNA (indicated with NC) and a vector expressing P65-UTR WT or P65-UTR MUT. Firefly luciferase activity was normalized to *Renilla* luciferase activity. The results are representative of three independent experiments. Data are presented as the means ± SD. * *P* < 0.05 vs. NC + P65-UTR-WT group (**j**, **k**)

As miR-155 and miR-99b were observed to be upregulated in BCG-infected macrophages, especially when Rv2346c was added, we hypothesized that they regulate NF-κB activation or p38 phosphorylation. Therefore, U937 and RAW264.7 cells were transfected with miR-155 or miR-99b mimics or with miR-155 or miR-99b inhibitors to evaluate their potential regulatory activities (Fig S8). The results showed that both miR-155 and miR-99b downregulated the expression of p65, and this effect was enhanced by their combined activity (Fig. 4a–d). When miR-155 and miR-99b were knocked down, the level of p65 was upregulated (Fig. 4e–h). Neither miR-155 nor miR-99b had an effect on p38 phosphorylation (Fig S9). This finding suggested that miR-155 and miR-99b function as negative regulators upstream of p65 but act downstream of p38 or had no obvious relationship with p38. To verify the hypothesis, the relationship between miRNAs (miR-155 and miR-99b) and p65 was analyzed using Miranda. The P65-UTR sequence was observed to contain a binding site for miR-155 but not for miR-99b (Fig. 4i). A dual-luciferase reporter assay was subsequently performed to verify the interaction between miRNAs (miR-155 and miR-99b) and p65. The results showed that miR-155 suppress luciferase expression, whereas miR-99b did not affect the luciferase expression significantly. Once the putative binding site is mutated, miR-155 failed to significantly downregulate the expression of luciferase (Fig. 4j, k and Fig S10). These findings indicated that a miR-155-binding site was present in the P65-UTR, and that miR-155 directly downregulated the expression of p65. The binding was specific, because the luciferase expression was not affected by the control miRNA.

**Fig. 5:**
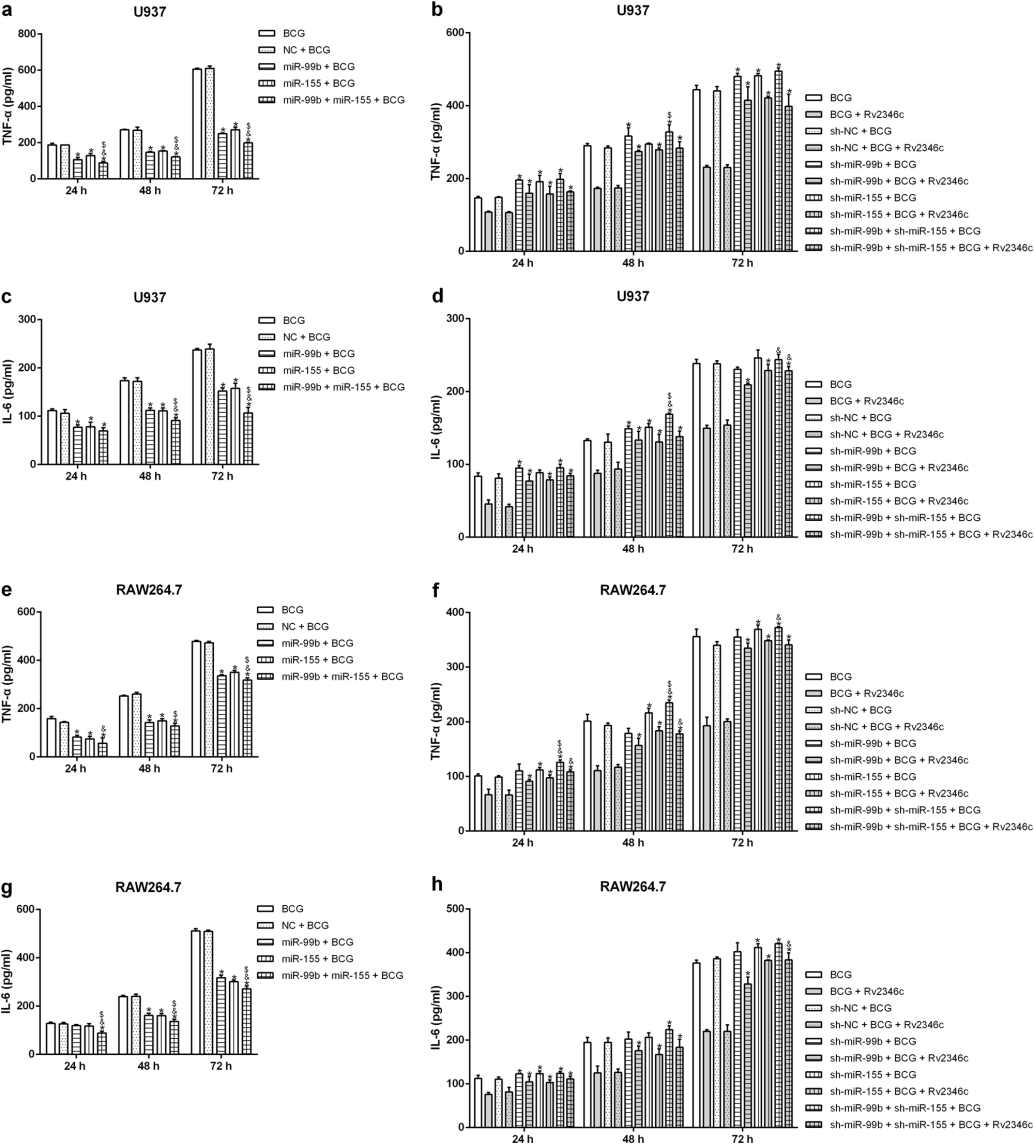
Effect of miR-155 and miR-99b on the production of TNF-α and IL-6. U937 and RAW264.7 cells were transfected with a negative control miRNA for mimic (indicated with NC), miR-155, or miR-99b mimics, both miR-155 and miR-99b mimics, a negative control miRNA inhibitor (indicated with sh-NC), a miR-155 inhibitor (indicated with sh-miR-155), a miR-99b inhibitor (indicated with sh-miR-99b), or both the miR-155 and miR-99b inhibitors. After 24 h post transfection, cells were infected with BCG (MOI 1/5) and cells in the BCG + Rv2346c group were also treated with Rv2346c (500 pg/ml). After incubating for 24, 48, or 72 h, cell culture supernatants were collected and detected with ELISA assay. The results are representative of three independent experiments. Data are presented as the means ± SD. *P < 0.05 vs. the negative control (NC group or sh-NC group) + BCG group (with or without Rv2346c treatment); &*P* < 0.05 vs. miR-99b/sh-miR-99b + BCG group (with or without Rv2346c treatment); $*P* < 0.05 vs. miR-155/sh-miR-155 + BCG group (with or without Rv2346c treatment)

To confirm the relationship between these miRNAs and p65, the effect of miR-155 and miR-99b on cytokine production was investigated. The results suggested that both miR-155 and miR-99b inhibited TNF-α and IL-6 production when overexpressed, and this effect was enhanced during their simultaneous overexpression. Knockdown of miR-155 and miR-99b upregulated TNF-α and IL-6 production, but this effect was not augmented when they were simultaneously knocked down (Fig. 5). This finding is in accordance with the impact of p65 on cytokine production, indicating that miR-155 and miR-99b negatively regulate p65, affecting cytokine production.

### p38 regulates miRNA expression, p65 level, and cytokine production

**Fig. 6:**
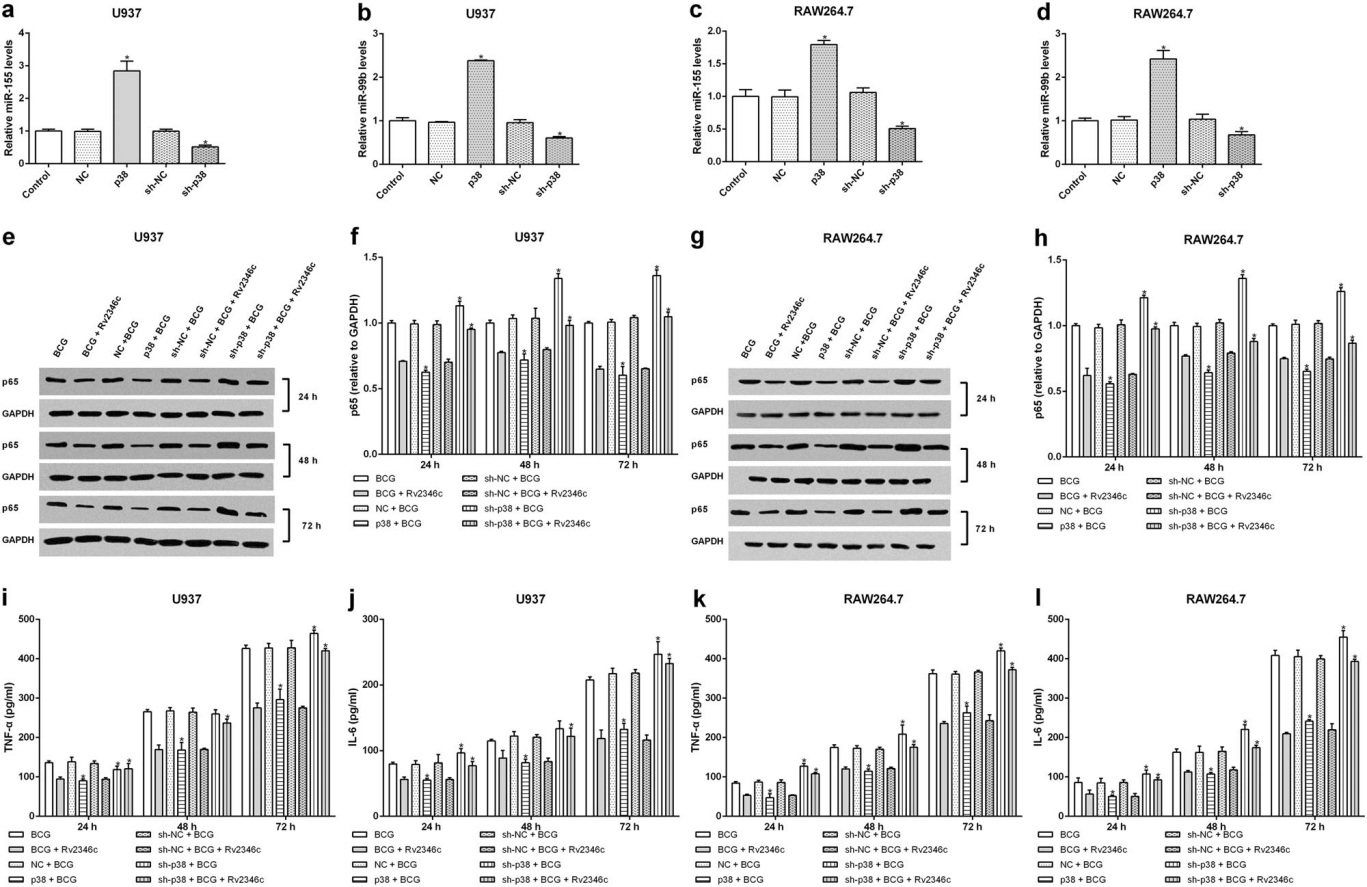
Effect of p38 on the levels of miR-155 and miR-99b, p65 expression and cytokine production. U937 and RAW264.7 cells were transfected with a control vector (indicated with NC), pcDNA3.1/p38 cDNA (indicated with p38), a nonspecific siRNA (indicated with sh-NC) or a specific siRNA directed against p38 (indicated with sh-p38). After 24 h post transfection, total RNA was extracted. miR-155 and miR-99b were detected via qRT-PCR. The results are representative of three independent experiments. Data are presented as the means ± SD. * *P* < 0.05 vs. the negative control (NC group or sh-NC group) (**a**–**d**). After 24 h post transfection, cells were infected with BCG (MOI 1/5) and cells in the BCG + Rv2346c group were also treated with Rv2346c (500 pg/ml). After incubating for 24, 48, or 72 h, total protein was extracted and cell culture supernatants were collected. Protein expression was detected via western blotting (**e**–**h**). The cytokines were detected via ELISA assay (**i**–**l**). The results are representative of three independent experiments. Data are presented as the means ± SD. *P < 0.05 vs. the negative control (NC group or sh-NC group) + BCG group (with or without Rv2346c treatment)

To identify the function of p38 in BCG-infected macrophages, U937 and RAW264.7 cells were transfected with pcDNA3.1/p38 cDNA or a specific siRNA directed against p38. The modified expression of p38 was verified by western blotting (Fig S11). The results showed that overexpression of p38 resulted in upregulation of miR-155 and miR-99b, and decreased the level of p65. In addition, knockdown of p38 caused the downregulation of miR-155 and miR-99b expression, and increased that of p65 (Fig. 6a–h). Overexpression of p38 also had a suppressive role on the production of TNF-α and IL-6. In contrast, knockdown of p38 upregulated the levels of TNF-α and IL-6 (Fig. 6i–l). These data indicated that Rv2346c enhanced BCG survival in macrophages via the p38/miRNA/NF-κB pathway.

### Rv2346c enhances the virulence of *Mtb* and inhibits the production of TNF-α and IL-6 in vivo

**Fig. 7:**
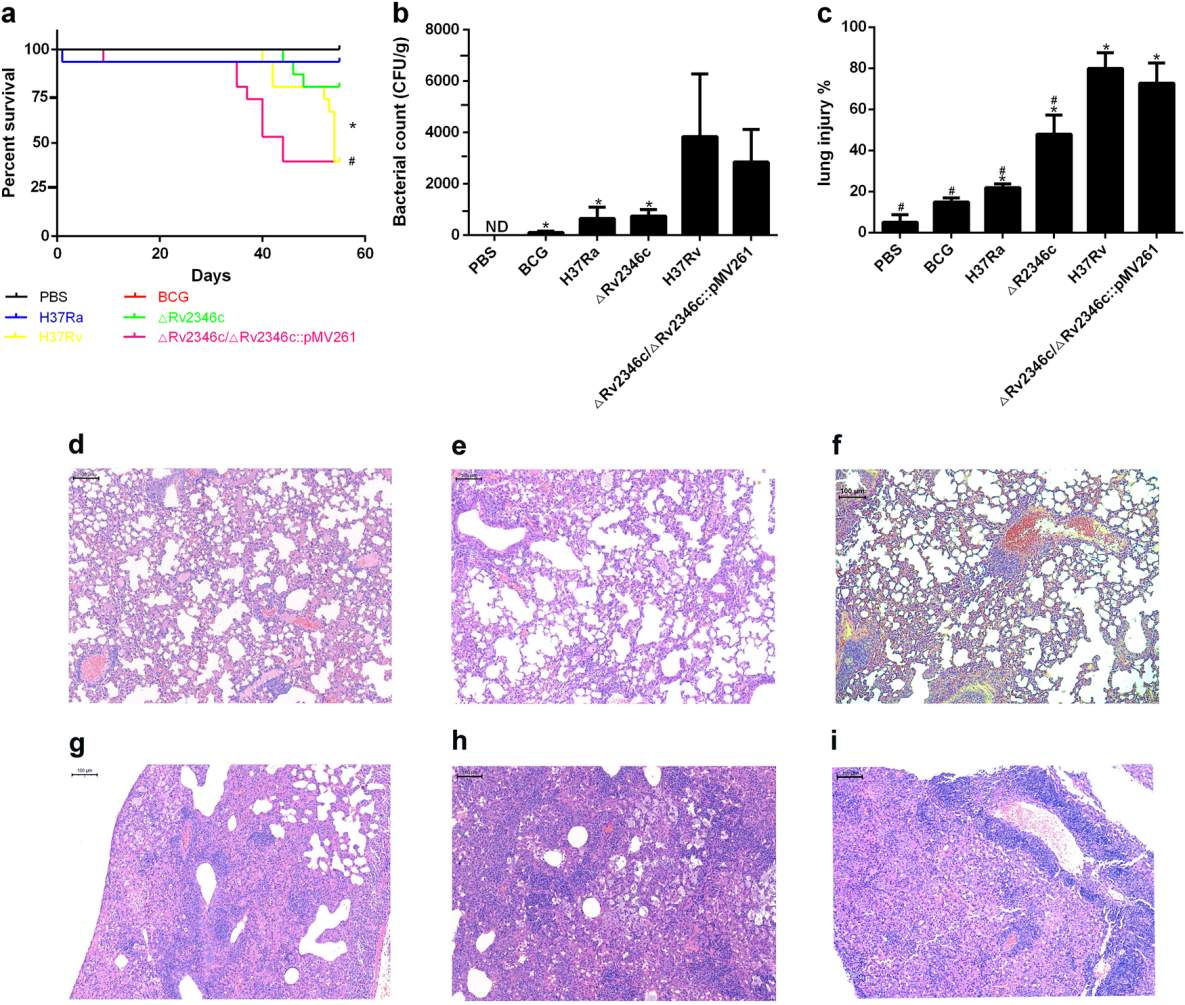
Effect of Rv2346c enhanced *Mtb* virulence. C57BL/6 mice were intratracheally infected with BCG, H37Ra, ΔRv2346c, H37Rv, or ΔRv2346c/ΔRv2346c::pMV261. Survival was monitored until 55 dpi. * *P* < 0.05 ΔRv2346c group vs. H37Rv group, #*P* < 0.05 ΔRv2346c group vs. ΔRv2346c/ΔRv2346c::pMV261 group (**a**). Lungs were collected at 35 dpi and used for a CFU assay or H&E staining. CFUs were normalized to grams of the lung tissue. *P < 0.05 vs. H37Rv group (**b**). Morphometric analysis of the total lesion was performed on H&E-stained sections of lung tissues. Scale bar represents 100 μm (**d**–**i**). Histograms show the mean percentage of lesion area within the total lung. *P < 0.05 vs. PBS group, #*P* < 0.05 vs. H37Rv group (**c**). In the survival assay, 15 mice were used for each group; the CFU results are representative of five independent experiments; the lung injury results are representative of three independent experiments. Data are presented as the means ± SD. **d** PBS; **e** BCG; f H37Ra; **g** ΔRv2346c; **h** H37Rv; **i** ΔRv2346c/ΔRv2346c::pMV261. dpi days post infection, ND not determined

To verify the function of Rv2346c in *Mtb* infection, C57BL/6 mice were intratracheally infected with the *Mtb* strains H37Rv, H37Ra, ΔRv2346c, or ΔRv2346c/ΔRv2346c::pMV261. The mouse mortality was observed for 55 days post infection (dpi), after which the mice were killed to obtain the lungs. Notably, ΔRv2346c-treated C57BL/6 mice showed a much lower mortality than the H37Rv- and ΔRv2346c/ΔRv2346c::pMV261-treated mice (Fig. 7a). In agreement with the corresponding survival curves, the bacterial load of ΔRv2346c-treated C57BL/6 mice was much lower than those observed for H37Rv- and ΔRv2346c/ΔRv2346c::pMV261-treated mice (Fig. 7b). Thus, the data demonstrated that Rv2346c augmented the virulence of *Mtb* in vivo. The lung tissues were collected at 35 dpi to observe *Mtb*-induced damage. Numerous inflammatory foci were observed in *Mtb*-infected lung parenchyma, except in mice treated with BCG (Fig. 7d–i). Histological lesions exhibited epithelioid and foamy macrophages and neutrophil clustering, as well as numerous islands of karyorrhectic debris surrounded by epithelioid macrophages and some multinucleated giant cells. Morphometric analysis of hematoxylin and eosin (H&E)-stained histological sections revealed that the lungs of ΔRv2346c-treated C57BL/6 mice were less injured than those of the H37Rv- and ΔRv2346c/ΔRv2346c::pMV261-treated mice (Fig. 7c).

**Fig. 8:**
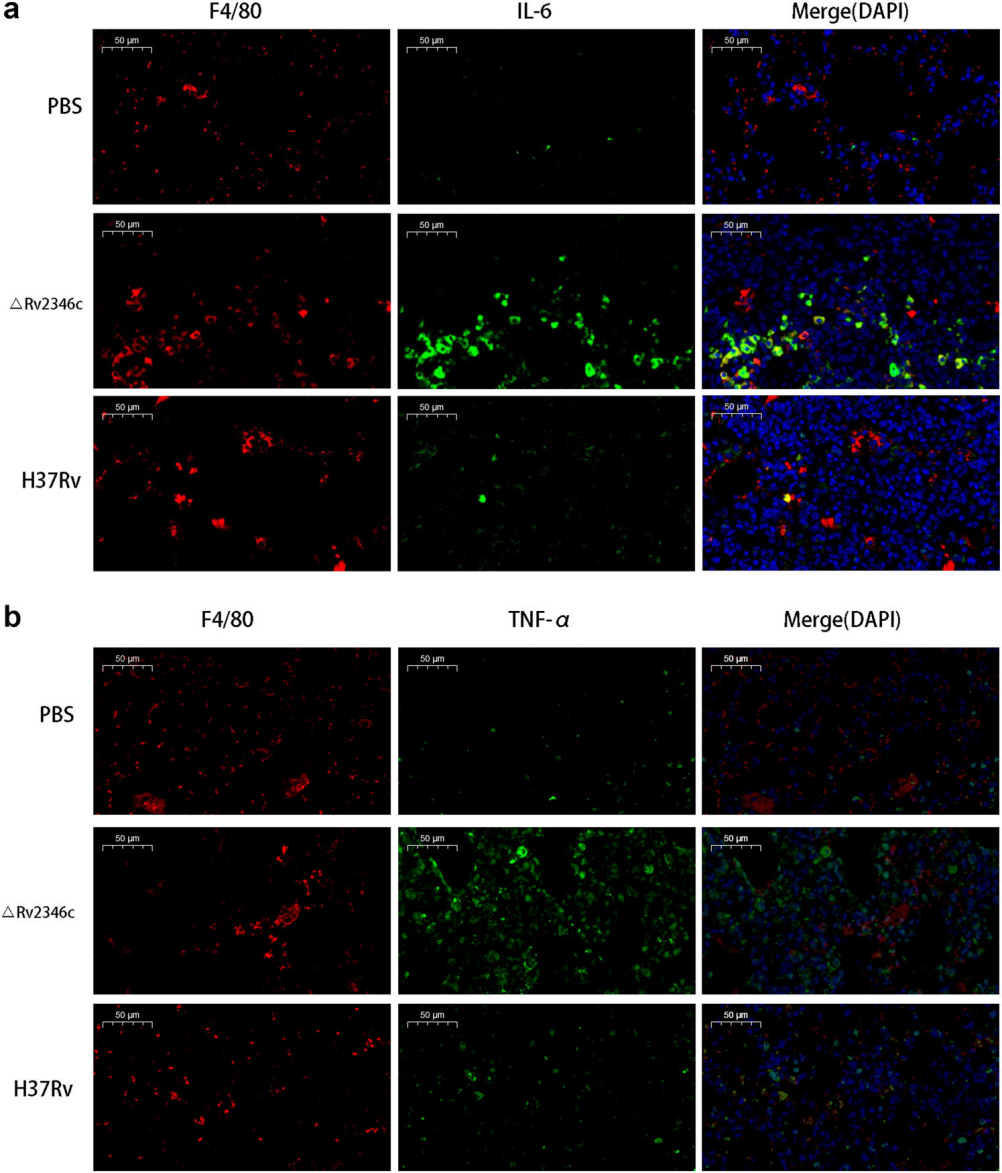
Effect of Rv2346c on cytokine production in vivo. C57BL/6 mice were intratracheally infected with ΔRv2346c or H37Rv. Lungs were collected at 35 dpi and used for immunofluorescence staining. All macrophages are indicated in red; IL-6 and TNF-α in green; nuclei in blue (DAPI). Scale bar represents 50 μm. dpi days post infection

Immunofluorescence analyses confirmed that mice treated with H37Rv and ΔRv2346c showed increasing amounts of macrophages infiltrated in the lungs and released greater levels of cytokines (TNF-α and IL-6) compared with mice treated with PBS (Fig. 8). In addition, the fluorescence intensity of TNF-α and IL-6 from mice treated with ΔRv2346c was higher than that observed from H37Rv-treated mice, indicating Rv2346c has a suppressive effect on the production of these cytokines, in accordance with the in vitro results.

## Discussion

Different genomic regions have been identified between virulent *Mtb* and avirulent BCG. A proteomic analysis of *Mtb* and BCG strains identified 13 and 8 unique proteins in *Mtb* H37Rv and BCG, respectively. Identifying differences in protein composition between virulent *Mtb* and attenuated vaccine strains is useful in the discovery of virulence factors and the development of targeted therapies^20^. Rv2346c, an ESAT-6-like protein, is only conserved in virulent *Mtb* strains and has been shown to promote bacterial persistence inside macrophages^14^. In this study, recombinant Rv2346c protein was produced by DNA template synthesis, PCR amplification, expression vector construction, and protein extraction and purification. The synthetic Rv2346c protein was verified by gene sequencing and western blot analyses and was subsequently used to investigate its importance in *Mtb* infection.

When transmitted via aerosolized droplets, its typical mode of transmission, *Mtb* is phagocytized by alveolar macrophages. Macrophages are considered to be the primary cell population on the frontline of host defenses^21^. Macrophages phagocytize mycobacteria by recognizing specific mycobacterial ligands, resulting in the subsequent activation of antimicrobial pathways. In turn, *Mtb* attempts to counteract the bactericidal activities of the host immunity and establish a niche for long-term survival within macrophages^4,22^. Human monocytic leukemia U937 cells and murine macrophage RAW264.7 cells infected with BCG were used to evaluate *Mtb* infection and were treated with recombinant Rv2346c protein to assess its function in *Mtb* pathogenesis. The results showed that Rv2346c inhibited the proliferation of BCG-infected macrophages and increased the survival of BCG in macrophages. Moreover, Rv2346c increased the survival of *Mtb* in vivo and aggravated lung injury. These observations indicate that Rv2346c may act as a virulence factor in *Mtb* infection.

TNF-α has a crucial role in the initial and long-term control of TB. Macrophages are induced to secrete TNF-α upon recognizing mycobacterial cell wall components. TNF-α subsequently activates macrophages, recruits them to the site of infection and participates in granuloma formation^23^. TNF-α secretion by *Mtb*-infected macrophages upregulates nitric oxide synthase 2 expression and induces the production of reactive nitrogen intermediates within phagolysosomes, mediating intracellular *Mtb* killing^15,24^. Neutralization of TNF-α leads to an increased susceptibility to TB and a lack of control of initial or chronic infections^23^. Mice deficient in TNF-α are susceptible to *Mtb* infection^25^. IL-6 also has a crucial role in protecting against *Mtb*, as infection with *Mtb* was observed to be lethal in IL-6-deficient mice^26^. Interestingly, a hypervirulent *Mtb* strain was shown to reduce the levels of proinflammatory cytokines, including TNF-α and IL-6^27^. In our study, the levels of TNF-α and IL-6 were upregulated in the supernatant of BCG-infected macrophages and were suppressed by Rv2346c, both in vitro and in vivo, consistent with the observed effect of Rv2346c on BCG survival.

The transcription factor NF-κB is essential for the induction expression of a variety of inflammatory genes in response to a range of pathogens and inflammatory cytokines. TNF-α is regulated by the NF-κB signaling pathway in the process of restricting bacterial survival in granulomas and aggregating bacteria and immune cells within the lung. NF-κB in resting cells remains latent in the cytoplasm by binding to IκB proteins. Binding of TNF to TNF receptor type1 leads to activation of the IκB kinase (IKK) and IKK-mediated phosphorylation of IκB proteins, which results in ubiquitination and proteasome-mediated degradation of IκB. This effect mediates the translocation of the p65 subunit of NF-κB, the accumulation of free NF-κB in the nucleus and induces the transcription of target genes. TNF-α is also included in these targeted genes^17,28^. In addition, IL-6 is reported to be induced in *Mtb*-infected macrophages through NF-κB activation^29^. The results showed that the transcriptional and translational expression of p65 increased during BCG infection and was inhibited by treatment with Rv2346c, indicating that p65 may be the signal molecule modulated by Rv2346c to inhibit the bactericidal activity of macrophages. This hypothesis was validated by the results obtained from the overexpression and knockdown of p65 in BCG-infected macrophages.

miRNAs are small non-coding endogenous RNA molecules that can regulate a wide range of biological processes via posttranscriptionally regulating gene expression. We performed a microarray assay to identify miRNAs associated with the process of BCG infection and that were affected by the Rv2346c treatment. In the microarray assay, miR-155 and miR-99b were the only miRNAs exhibiting a greater than twofold increase in both RAW264.7 and U937 cells after treatment with Rv2346c during BCG infection. These miRNAs have also been reported to affect the production of cytokines associated with *Mtb* infection. miR-155 has been confirmed as a multifunctional miRNA involved in a variety of biological processes, including infection, inflammation and immunity^9,30^. Dysregulation of miR-155 has been reported in human macrophages that are infected by different *Mycobacterium* species^9^. miR-155 has been reported to be the most highly expressed miRNA in murine macrophages and bone marrow-derived macrophages upon *Mtb* infection^31,32^. Furthermore, miR-155 is associated with TNF-α and IL-6 production^32,33^. The expression of another miRNA, miR-99b, has been reported to result in significant reductions in *Mtb* loads and notable upregulation of proinflammatory cytokines, such as TNF-α and IL-6, when its expression is blocked^32,34^. However, the mechanism underlying this miRNA-mediated effect remains to be determined. To identify miRNAs associated with *Mtb* infection, a microarray assay was performed using BCG-infected macrophages. The results showed that levels of miR-155 and miR-99b were significantly higher in BCG-infected macrophages treated with Rv2346c compared with untreated cells. The results of a qRT-PCR showed that miR-155 and miR-99b were upregulated in macrophages infected with BCG, which were further increased in cells treated with Rv2346c. Further investigation suggested that miR-155 and miR-99b inhibited the expression of p65 and cytokine production, and that a miR-155-binding site is present in the P65-UTR, indicating that miR-155 and miR-99b are negative regulators of p65.

Previous studies have shown that mycobacterial infection acts through TLRs to trigger MAPK pathways, leading to activation of transcription factors, including NF-κB^35,36^. TNF-α production was shown to be induced through a signaling pathway that requires activation of p38 MAPKs in *Mtb*-infected macrophages^37,38^. In addition, a TLR2 or TLR4 deficiency suppressed the activation of p38 and NF-κB, and negatively regulated IL-6 synthesis in H37Rv-infected neutrophils^39^. Activation of p38 and NF-κB mediates IL-6 production in macrophages^40^. However, it was also reported that the inhibition of p38 in macrophages increases the expression of TNF-α and IL-6^41^. The results of the present study showed that the phosphorylation of p38 was upregulated due to BCG infection, indicating that p38 activation promotes antibacterial activity in macrophages. However, p38 phosphorylation was further augmented during Rv2346c intervention, the role of p38 activation during BCG infections is complex and unclear. Further investigation revealed that p38 upregulated the levels of miR-155 and miR-99b, and inhibited the expression of p65 and cytokine production, although changes in p65, miR-155, and miR-99b expression did not notably affect p38 phosphorylation. Thus, we speculate that p38 acts as a positive regulator upstream of miR-155 and miR-99b.

According to our results, we conclude that macrophages produce TNF-α and IL-6 to mediate killing of BCG through activation of NF-κB. Rv2346c suppress the secretion of TNF-α and IL-6, and facilitates the survival of BCG through the p38/miR-155 and miR-99b/NF-κB-pathways. The results of this investigation suggest that Rv2346c acts as a crucial virulence factor during *Mtb* infection and has potential use as a target for anti-TB therapy.

## Materials and methods

### Production of recombinant Rv2346c protein

A DNA fragment encoding Rv2346c was obtained from GenScript (Nanjing, China) and the oligonucleotide primers were designed with Primer 5.0 (Premier, Canada) (Table S1). The DNA template and primers were synthesized by GenScript and PCR was performed with the template described above. The thermocycling program used was as follows: 94 °C for 5 min, followed by 30 cycles of 96 °C for 25 s, 58 °C for 25 s, and 72 °C for 1 min. The PCR product was cut and purified via DNA gel extraction kit (AXYGEN, NY, USA). The purified PCR product and pET-30a( + ) (Novagen, Germany) were digested with NdeI and HindIII (NEB) (Thermo Scientific, DE, USA) and ligated together with T4 DNA ligase (Thermo Scientific). The pET-30a( + ) vector containing the Rv2346c-encoding gene was transformed by electroporation into *E. coli* strain BL21(DE3) (Novagen) and the cells were subsequently grown on Luria-Bertani (LB) agar plates containing 50 µg/ml kanamycin at 37 °C overnight. The positive clones were selected and confirmed by sequencing. The confirmed plasmids were transformed into *E. coli* BL21(DE3) and plated onto solid medium, after which a single colony was selected and cultivated in LB medium in a shaking incubator. When the OD_600_ reached 0.6, cells were induced with isopropyl-β-d-thiogalactoside (1 mmol/L). Next, the culture was centrifuged and cells were resuspended and lysed by sonication. The sonicated sample was centrifuged and the supernatant of the cell lysate was applied to a Ni-IDA resin (GenScript). The protein was subsequently eluted and the eluate was collected for SDS-polyacrylamide gel electrophoresis analysis. A protein with the predicted mass of Rv2346c was concentrated through ultrafiltration (molecular weight cutoff 10 kDa). The concentration of the protein was determined by the Bradford method. Subsequently, a western blotting was performed to examine the purified recombinant Rv2346c protein via the N-His Tag (His Tag antibody; GenScript).

### Cell culture

U937 cells (American Type Culture Collection (ATCC), Manassas, VA, USA), a human monocytic leukemia cell line, were cultured in RPMI-1640 medium (Gibco BRL, Grand Island, NY, USA) supplemented with 10% fetal bovine serum (FBS, Gibco BRL), penicillin (100 U/ml), streptomycin (100 µg/ml), and l-glutamine (2 mM), and were maintained at 37 °C under a humidified atmosphere containing 5% CO_2_.

RAW264.7 cells (ATCC), a murine macrophage cell line, were cultured in DMEM medium (Gibco BRL) supplemented with 10% FBS, penicillin (100 U/ml), streptomycin (100 µg/ml), and l-glutamine (2 mM), and were maintained at 37 °C under a humidified atmosphere containing 5% CO_2_.

### Bacterial culture

The BCG Beijing strain developed from BCG Denmark was provided by the Jiangsu Provincial Center for Disease Control and Prevention. BCG was grown on Sauton’s medium. After culturing for 3 weeks, bacteria were collected and resuspended in cell culture medium without antibiotics. The solution was adjusted to an OD_600_ of 0.5 (~10^7^ bacteria/ml). The bacterial suspension was used to infect cells at the indicated MOI.

*Mtb* H37Rv (ATCC, no.27294), H37Ra (ATCC, no.25177), the Rv2346c-deleted strain (ΔRv2346c) (Shanghai Gene-optimal Science & Technology, Shanghai, China) and the Rv2346c-gene complemented strain (ΔRv2346c/ΔRv2346c::pMV261) (Shanghai Gene-optimal Science & Technology) were grown in Middlebrook 7H9 broth (Difco, Detroit, MI, USA) supplemented with 0.2% glycerol, 0.05% Tween 80, and 10% Middlebrook OADC for 4–6 weeks. Mid-log phase cultures were harvested and stored at − 80 °C. Before infecting animals, stock solutions of *Mtb* were thawed, washed, and diluted in sterile distilled water to a specific concentration.

### Transfection

The fragments encoding the sequences of the p65 and p38 alleles were obtained from GenScript and cloned into pcDNA3.1 (Invitrogen, Carlsbad, CA, USA). U937 and RAW264.7 cells were transiently transfected with pcDNA3.1/p65 cDNA, pcDNA3.1/p38 cDNA, control pcDNA3.1, miR-155 mimic (GenePharma, Shanghai, China), miR-99b mimic (GenePharma), or a negative control (NC) miRNA (GenePharma) using Lipofectamine 3000 (Invitrogen) according to the manufacturer’s instructions. A control pcDNA3.1 or an NC miRNA was used as a NC. Cells were incubated for 24 h at 37 °C before being used for further analysis.

siRNAs targeting NF-κB p65 subunit mRNA or p38 mRNA, a random non-coding siRNA, a miR-155 inhibitor, a miR-99b inhibitor and an NC mRNA were synthesized by GenePharma. U937 and RAW264.7 cells were transfected with the above siRNAs or miRNAs using Lipofectamine 3000 according to the manufacturer’s instructions. A non-coding siRNA or an NC miRNA was used as a NC. The cells were exposed to BCG and recombinant Rv2346c protein at 24 h post transfection for additional assays. The corresponding sequences of these RNAs are shown in Table S2.

### Proliferation assay

U937 or RAW264.7 cells were seeded at 5000 cells per well in 96-well plates. After incubating overnight, the BCG-infected groups were infected with BCG at the indicated MOIs (cell/bacillus: 1/1, 1/5, 1/10, 1/50). The BCG + Rv2346c groups were infected with BCG (MOI: 1/5) and treated with Rv2346c at the indicated concentrations (50, 100, 500, and 1000 pg/ml). For the control group, cells were treated with an equal volume of medium. The final volume was adjusted to 100 µl per well. After incubating for 24, 48, 72, or 96 h, the proliferation assay was performed using a CCK-8 kit (Dojindo Laboratories, Kumamoto, Japan) according to the manufacturer’s protocol.

### Bacterial enumeration

U937 or RAW264.7 cells were plated in 24-well plates (1 × 10^5^ cells per well) and cultured overnight. Cells in the BCG-infected group were infected with BCG (MOI: 1/5), whereas cells in the BCG + Rv2346c group were infected with BCG (MOI 1/5) and treated with recombinant Rv2346c protein (500 pg/ml). Cells were washed with warm phosphate-buffered saline after 4 h to remove non-ingested mycobacteria. Subsequently, after incubating for 24, 48, or 72 h, the infected cells were washed and lysed. The lysates were serially diluted (1:100), and 100 μl of the suspensions were plated on Sauton’s medium. CFUs was counted after incubation at 37 °C for 4 weeks.

### ELISA assay

Cell-free supernatants were collected and used to evaluate the concentrations of TNF-α and IL-6 with a human TNF-α ELISA kit (R&D Systems, Minneapolis, MN, USA), a human IL-6 ELISA kit (R&D Systems), a mouse TNF-α ELISA kit (R&D Systems), and a mouse IL-6 ELISA kit (R&D Systems) according to the manufacturer’s instructions.

### Western blotting

U937 and RAW264.7 cells were infected with BCG (MOI 1/5) and cells in the BCG + Rv2346c groups were treated with Rv2346c (500 pg/ml). After incubating for 24, 48, or 72 h, the cells were collected and lysed. The lysates were centrifuged, denatured, applied to an SDS-polyacrylamide gel for electrophoresis and transferred to a polyvinylidene fluoride membrane. The membranes were then blocked with 5% non-fat dry milk at room temperature for 1 h, followed by an incubation with primary antibodies at 4 °C overnight. The primary antibodies included an anti-NF-κB p65 (Abcam, MA, USA), an anti-NF-κB p65 (phospho S536) antibody (Abcam), an anti-p38 antibody (Abcam), an anti-p38 (phospho Y182) antibody (Abcam), and an anti-glyceraldehyde-phosphate dehydrogenase (GAPDH) antibody (Abcam). After washing, the membranes were incubated with the appropriate horseradish peroxidase-conjugated secondary antibody at room temperature for 2 h. The results were visualized via chemiluminescence using an ECL kit (Thermo scientific) and photographed with a Tanon MultiImager. The density of the immunoreactive bands was measured using ImageJ; GAPDH was used as an internal control.

### Microarray data analysis

U937 or RAW264.7 cells were infected with BCG (MOI 1/5) with or without the Rv2346c (500 pg/ml) treatment. After incubating for 24 h, cells were collected for total RNA extraction. RNA labeling was performed with a miRNA Complete Labeling and Hyb Kit (Agilent Technologies, Santa Clara, CA, USA) according to the manufacturer’s protocol. Each slide was hybridized with 100 ng of Cy3-labeled RNA in a hybridization oven set at 55 °C, 20 r.p.m. for 20 h. After hybridization, slides were washed in staining dishes with a Gene Expression Wash Buffer Kit (Agilent Technologies), scanned by an Agilent Microarray Scanner, and analyzed using Feature Extraction 10.7 (Agilent Technologies). Raw data were normalized by a Quantile algorithm using Gene Spring 12.6 (Agilent Technologies). The pairwise expression fold change was calculated after merging the spots with same Agilent probe ID (Agilent Human miRNA (8*60 K) V19.0, design ID: 46064; Agilent Mouse miRNA (8*60 K), design ID:58698). Differentially expressed genes were defined as those exhibiting a |fold change| > 2.

### Quantitative RT-PCR

U937 and RAW264.7 cells were infected with BCG (MOI 1/5) with or without the Rv2346c (500 pg/ml) treatment. After incubating for 24, 48, or 72 h, total RNA was extracted from the cells using a Total RNA Extraction Kit (Generay Biotech, Shanghai, China). gDNA ERASER (Takara, Shiga, Japan) was used to eliminate DNA when extracting RNA. RNA was reverse-transcribed into cDNA using a RevertAid First Strand cDNA synthesis Kit (Thermo Scientific Fisher, Waltham, MA, UK) and the thermocycling program used was as follows: 37 °C for 60 min and 85 °C for 5 min. Amplification was conducted using a Stepone plus Real-Time System with a SYBR Premix ExTM Taq (Tli RNaseH Plus) Kit (TaKaRa, Tokyo, Japan) under the following thermocycling conditions: 50 °C for 3 min and 95 °C for 15 min, followed by 40 cycles of 95 °C for 10 s, 60 °C for 20 s, and 72 °C for 30 s. mRNA expression was calculated using the 2^−ΔΔCt^ method. All values were normalized to the housekeeping gene GAPDH. The PCR primers used are listed in Table S1.

Total RNA for miR-99b, miR-155, and U6 detection was extracted with a Total RNA Extraction Kit. Reverse transcription and PCR was performed using a Bulge-Loop^TM^ miRNA qRT-PCR Starter Kit, a Bulge-Loop™ miRNA qRT-PCR Primer Set, and a U6 snRNA qPCR Primer Set (RiboBio, Guangzhou, China) according to the manufacturer’s instructions. miRNA expression was quantified using the 2^−ΔΔCt^ method. U6 was used as an internal control.

### Dual-luciferase reporter assay

The wild-type (WT) and mutant (MUT) 3′-untranslated region (3′-UTR) fragment of p65 (human/mouse) was inserted into the pGL3-basic vector (firefly luciferase; Promega, Madison, WI, USA), which was obtained from General Biosystems (Anhui, China). U937 or RAW264.7 cells were co-transfected with p65-UTR-WT or p65-UTR-MUT plasmids along with miR-155 or miR-99b mimics or scramble oligonucleotides using Lipofectamine 3000. A reporter vector carrying the WT or MUT sequences of p65-UTR was assayed for luciferase expression using the Dual-Luciferase® Reporter Assay System (Promega) following the manufacturer’s instructions. For data analysis, firefly luciferase activity was normalized to the corresponding *Renilla* luciferase activity.

### Infection of mice

Six- to 8-week-old female C57BL/6 mice (Cavens Lab Animal Company, Changzhou, China) were housed in a bio-safety level III animal facility under specific pathogen-free conditions. All animal experimental procedures were approved by the Institutional Animal Ethics Committee of the Second Affiliated Hospital of Nanjing Medical University (No.2014KY050) and were carried out in strict accordance with the Nanjing Medical University’s guidelines for their care and use. Mice were anesthetized using an intraperitoneal injection of 4% chloral hydrate and were inoculated intratracheally with each *Mycobacterium* strain at a dose of 2 × 10^6^ CFU per mouse. The survival of mice was monitored daily from the day of infection to 55 dpi.

### Bacterial load assessment in the lung

To determine the bacterial loads in the lungs, the mice were killed at 35 dpi. Lungs were removed aseptically, weighed, and homogenized. Serial dilutions of tissue homogenates were plated onto Middlebrook 7H11 agar. Bacterial CFUs were enumerated after 4–6 weeks of incubation at 37 °C.

### Histology examination

Lung lobes were collected at 35 dpi, fixed with 4% paraformaldehyde overnight, and embedded in paraffin. H&E-stained tissues were assessed via a pathology analysis. Lung injury was estimated by the percentage of the lesion area in the total lung area using an ImagePro macro.

### Immunofluorescence detection

Immunofluorescence detection of TNF-α and IL-6 was performed using tissue sections that were dewaxed and rehydrated. Antigen retrieval was performed using a proteinase K and hot citric acid buffer treatment as needed. The restored sections were incubated with primary antibodies overnight at 4 °C, including anti-F4/80 (Abcam), anti-TNF-ɑ (Abcam), and anti-IL-6 (Abcam) antibodies. Macrophages were observed using an F4/80 antigen. Next, tissue sections were stained with Alexa Fluor® 488-conjugated or Alexa Fluor® 647-conjugated secondary antibodies (Abcam) for 1 h at room temperature and mounted with SlowFade® Gold Antifade with 4’,6-diamidino-2-phenylindole (Invitrogen). Images were taken with a Leica Microsystems Ltd microscope and were analyzed using ImagePro Plus 6.

### Statistical analysis

All the presented results were confirmed in three independent experiments. The Student’s *t*-test was used to compare two groups and one-way analysis of variance followed by Least Significant Difference (LSD) was used to compare multiple groups. Survival curves was analyzed using the Log-rank test. Statistical analyses were performed using SPSS 20.0 (IBM SPSS, Armonk, NY, USA). *P* *<* 0.05 was considered to be significantly different.

### Data availability

All data generated or analyzed during this study are included in this published article (and its Supplementary Information files).

## Electronic supplementary material

Supplemental Tables

S1 Figure

S2 Figure

S3 Figure

S4 Figure

S5 Figure

S6 Figure

S7 Figure

S8 Figure

S9 Figure

S10 Figure

S11 Figure
