# Supplementary material for: Rv2346c enhances mycobacterial survival within macrophages by inhibiting TNF-α and IL-6 production via the p38/miRNA/NF-κB pathway
Source: Emerg Microbes Infect. 2018 Sep 19;7:158. doi: 10.1038/s41426-018-0162-6 (PMC6145905; doi:10.1038/s41426-018-0162-6)
Supplement: Supplementary file 1 — Supplemental Tables [file 41426_2018_162_MOESM1_ESM.docx]

**Supporting information**

**S1 Table A list of primers used for PCR**

| Primer | Sequence |
| --- | --- |
| Rv2346c-Forward | 5’-AACTTTAAGAAGGAGATATA-3’ |
| Rv2346c-Reverse | 5’-GCGGCCGCACTCGAGCAC-3’ |
| Human-p65-Forward | 5’-CTGTCCTTTCTCATCCCATCTT-3’ |
| Human-p65-Reverse | 5’-TCCTCTTTCTGCACCTTGTC-3’ |
| Mouse-p65-Forward | 5’-GCTCAAGATCTGCCGAGTAAA-3’ |
| Mouse-p65-Reverse | 5’-GTCCCGTGAAATACACCTCAA-3’ |
| Human-GAPDH-Forward | 5’-CAGGGCTGCTTTTAACTCTGGTAA-3’ |
| Human-GAPDH-Reverse | 5’-GGGTGGAATCATATTGGAACATGT-3’ |
| Mouse-GAPDH-Forward | 5’-CCAGTATGACTCCACTCACG-3’ |
| Mouse-GAPDH-Reverse | 5’-GACTCCACGACATACTCAGC-3’ |

**S2 Table A list of RNA for transfection**

| RNA | sequence |
| --- | --- |
| p65-Homo-specific siRNA | 5’-GCCCUAUCCCUUUACGUCATT-3’ |
| p65-Mus-specific siRNA | 5’-AGUCCCUGUCUGCACCUGUTT-3’ |
| p38-Homo-specific siRNA | 5’-GCAUAAUGGCCGAGCUGUUTT-3’ |
| p38-Mus-specific siRNA | 5’-GACUGUGAGCUCAAGAUUCTT-3’ |
| A non-specific siRNA | 5’-UUCUCCGAAGGUGUCACGUTT-3’ |
| miR-155 mimic (human/mouse) | 5’-UUAAUGCUAAUCGUGAUAGGGGU-3’ |
| miR-99b mimic (human/mouse) | 5’-CACCCGUAGAACCGACCUUGCG-3’ |
| A NC miRNA for mimic (human/mouse) | 5’-UUCUCCGAACGUGUCACGUUU-3’ |
| Human-miR-155 inhibitor | 5’-ACCCCUAUCACGAUUAGCAUUAA-3’ |
| Mouse-miR-155 inhibitor | 5’-ACCCCUAUCACAAUUAGCAUUAA-3’ |
| Human-miR-99b inhibitor | 5’-CGCAAGGUCGGUUCUACGGGUG-3’ |
| Mouse- miR-99b inhibitor | 5’-CGCAAGGUCGGUUCUACGGGUG-3’ |
| a NC miRNA for inhibitor | 5’-CAGUACUUUUGUGUAGUACAA-3’ |

Abbreviation: NC: negative control
