# Supplementary figures and images for: Rv2346c enhances mycobacterial survival within macrophages by inhibiting TNF-α and IL-6 production via the p38/miRNA/NF-κB pathway
Source: Emerg Microbes Infect. 2018 Sep 19;7:158. doi: 10.1038/s41426-018-0162-6 (PMC6145905; doi:10.1038/s41426-018-0162-6)

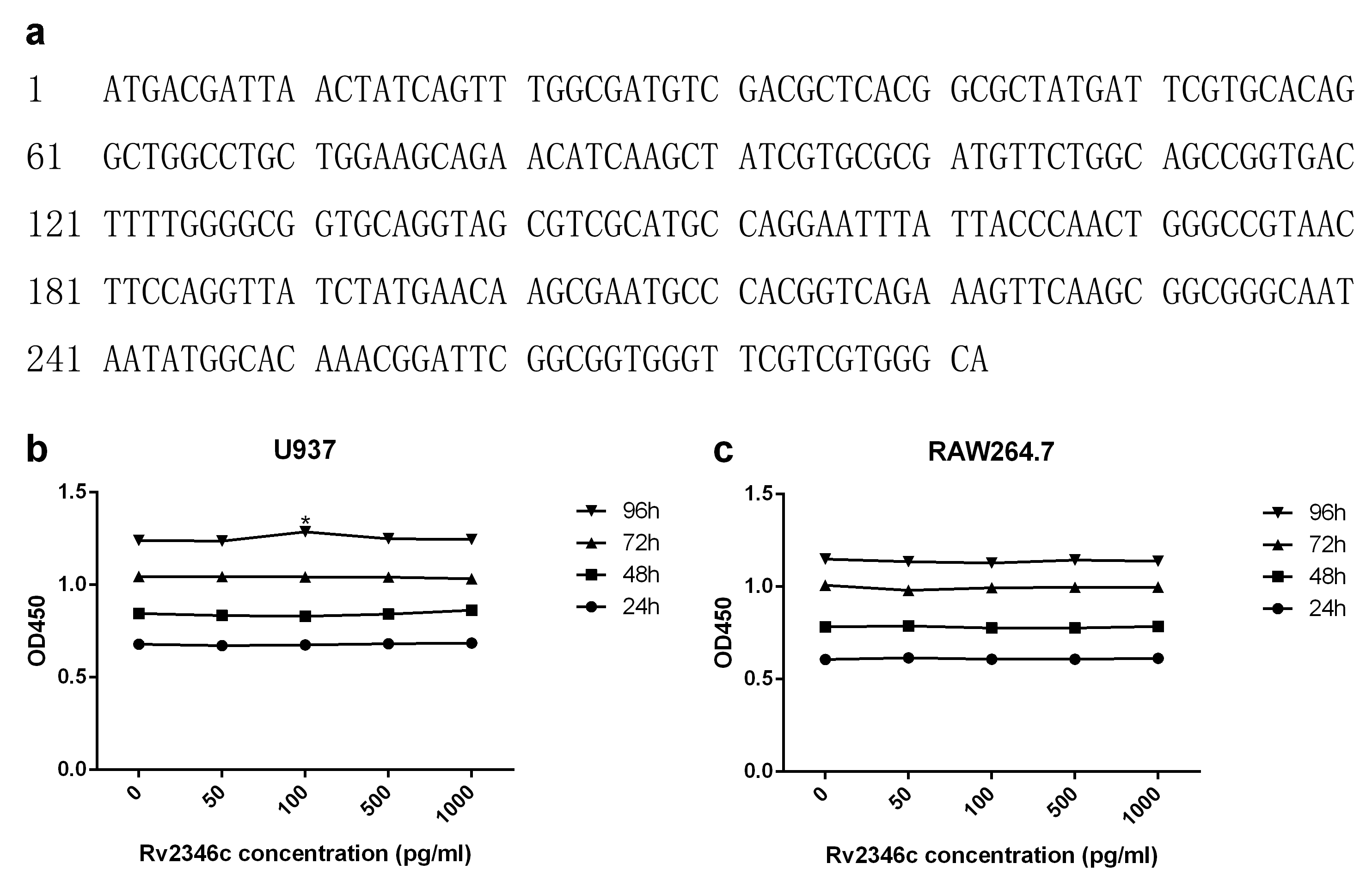

Supplement: Supplementary file 2 — S1 Figure [file 41426_2018_162_MOESM2_ESM.tif]

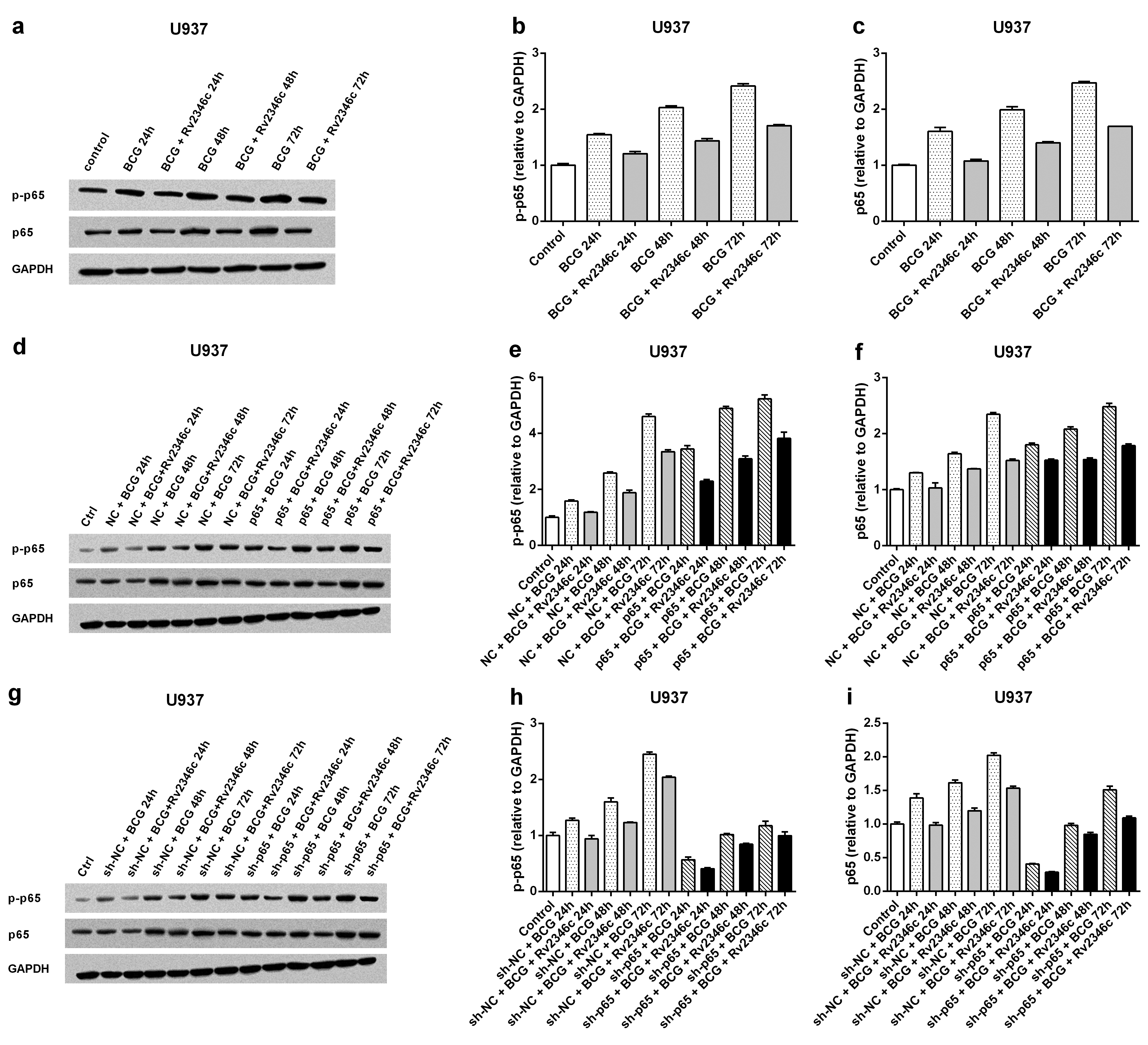

Supplement: Supplementary file 3 — S2 Figure [file 41426_2018_162_MOESM3_ESM.tif]

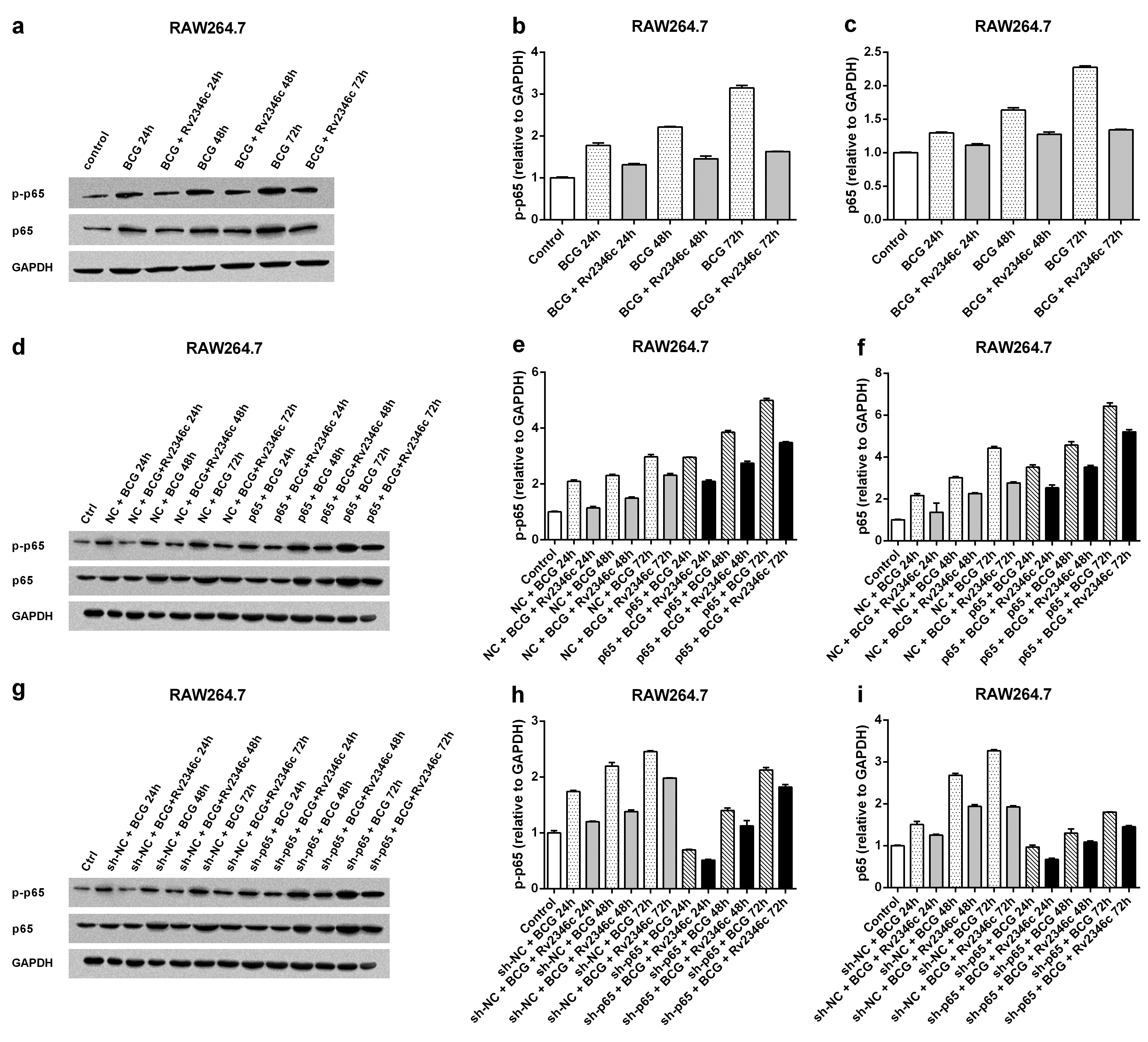

Supplement: Supplementary file 4 — S3 Figure [file 41426_2018_162_MOESM4_ESM.tif]

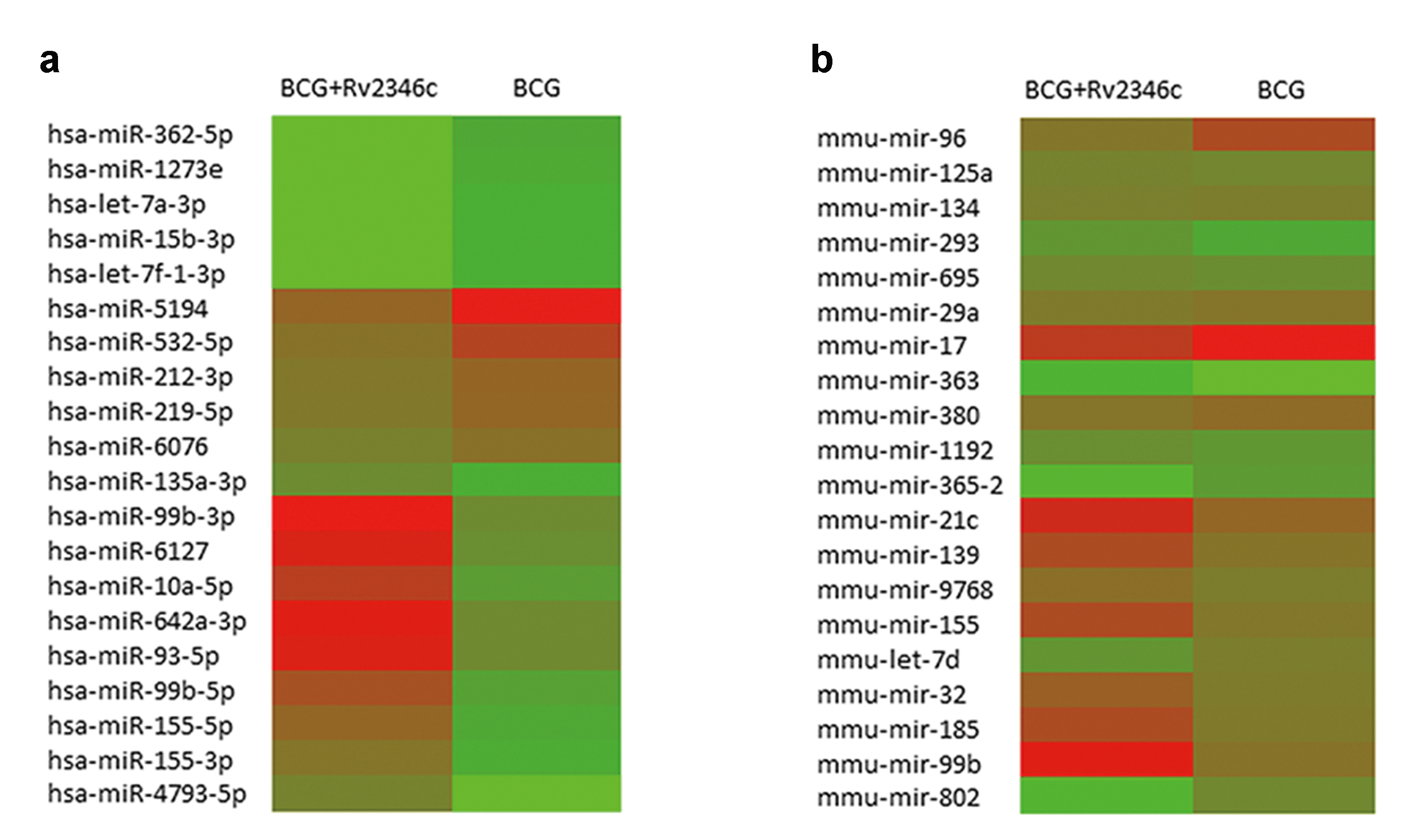

Supplement: Supplementary file 5 — S4 Figure [file 41426_2018_162_MOESM5_ESM.tif]

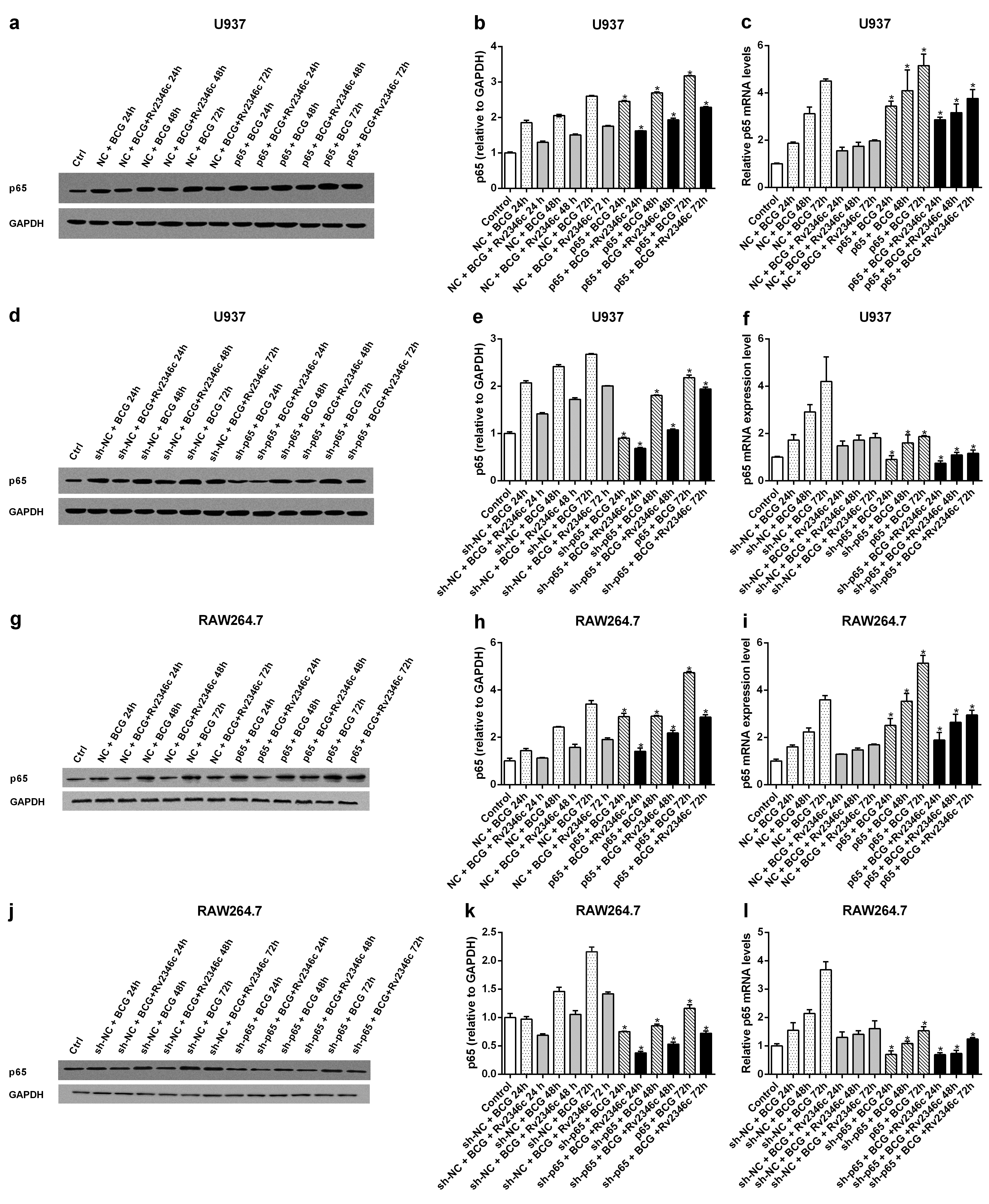

Supplement: Supplementary file 6 — S5 Figure [file 41426_2018_162_MOESM6_ESM.tif]

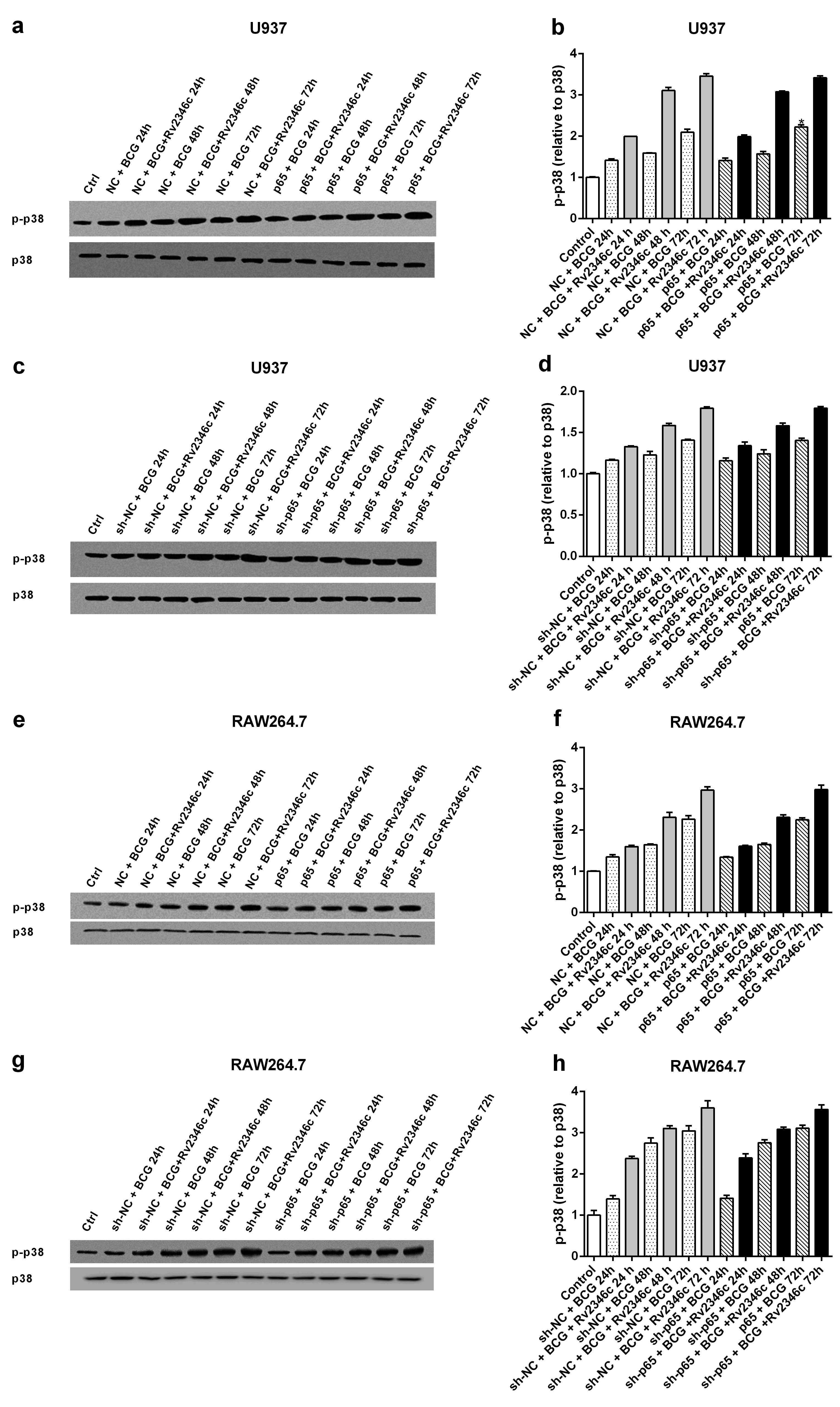

Supplement: Supplementary file 7 — S6 Figure [file 41426_2018_162_MOESM7_ESM.tif]

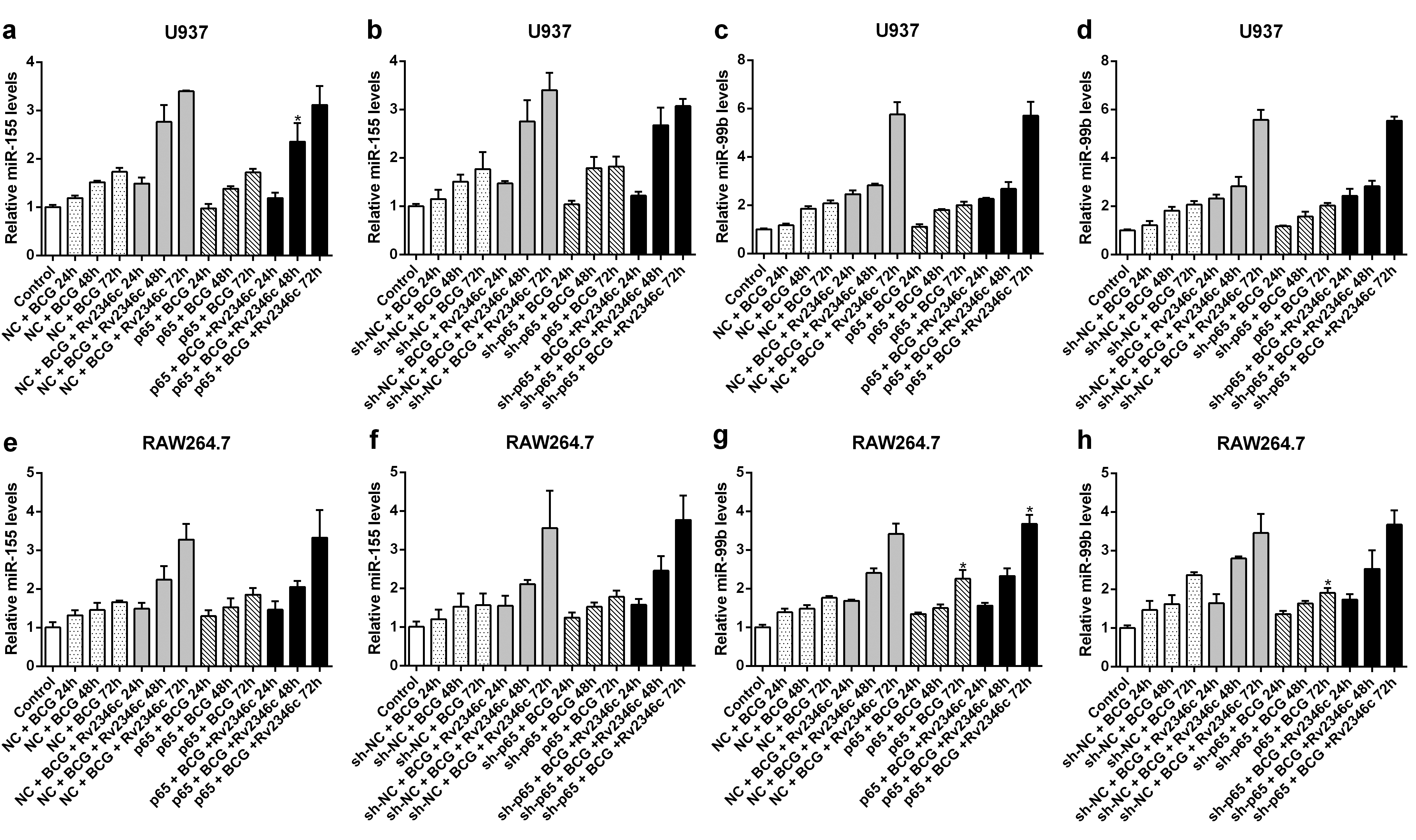

Supplement: Supplementary file 8 — S7 Figure [file 41426_2018_162_MOESM8_ESM.tif]

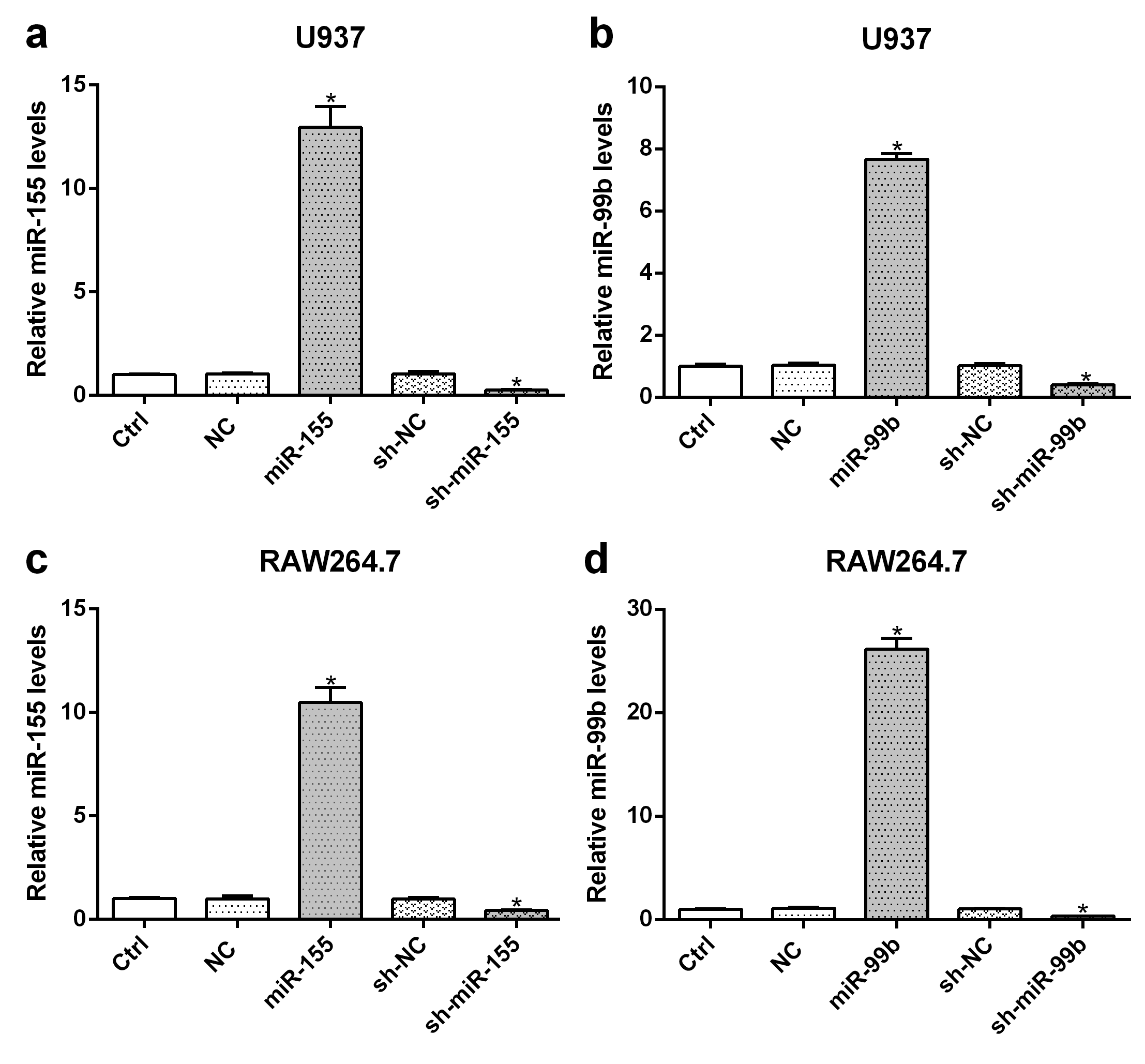

Supplement: Supplementary file 9 — S8 Figure [file 41426_2018_162_MOESM9_ESM.tif]

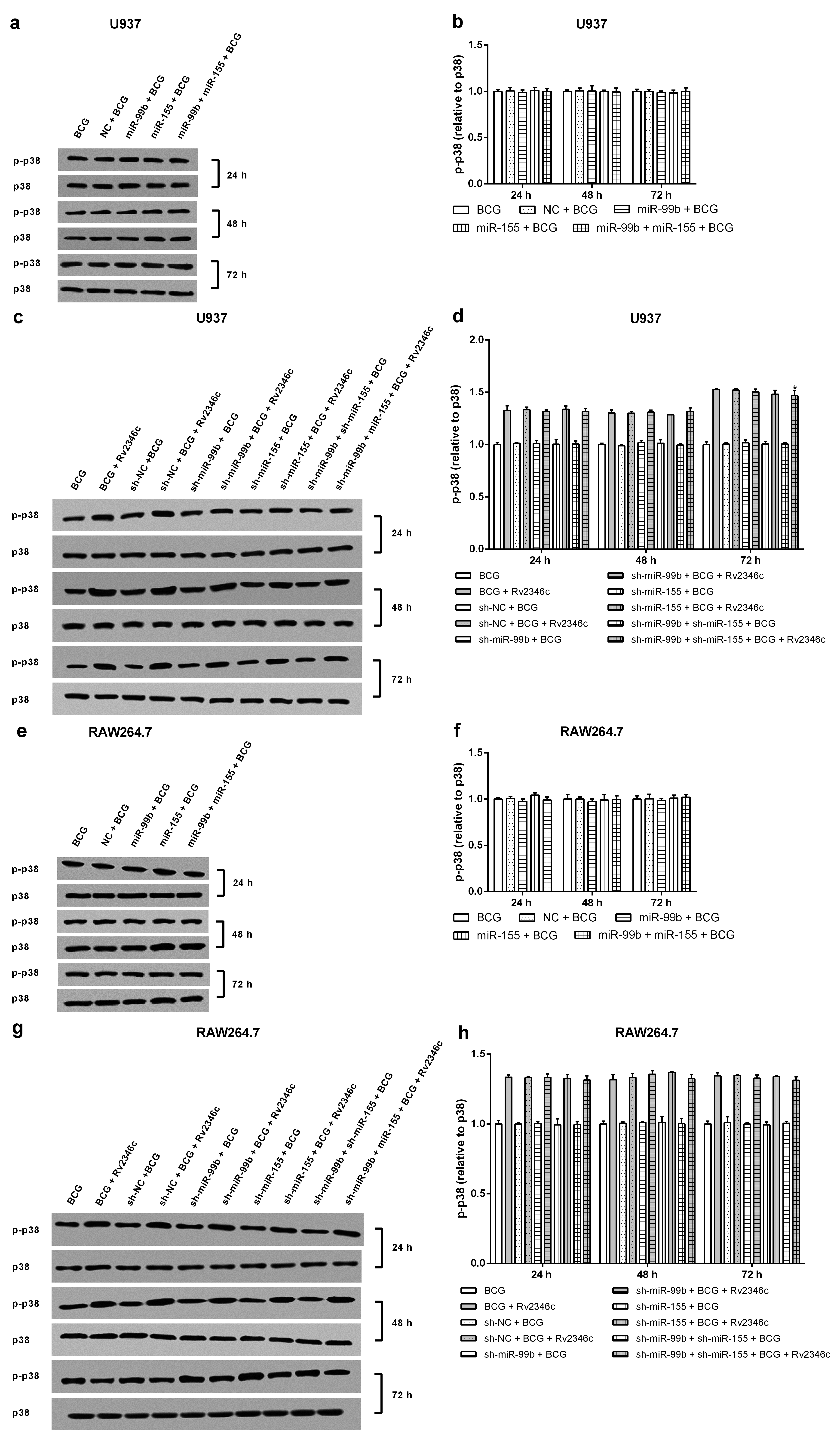

Supplement: Supplementary file 10 — S9 Figure [file 41426_2018_162_MOESM10_ESM.tif]

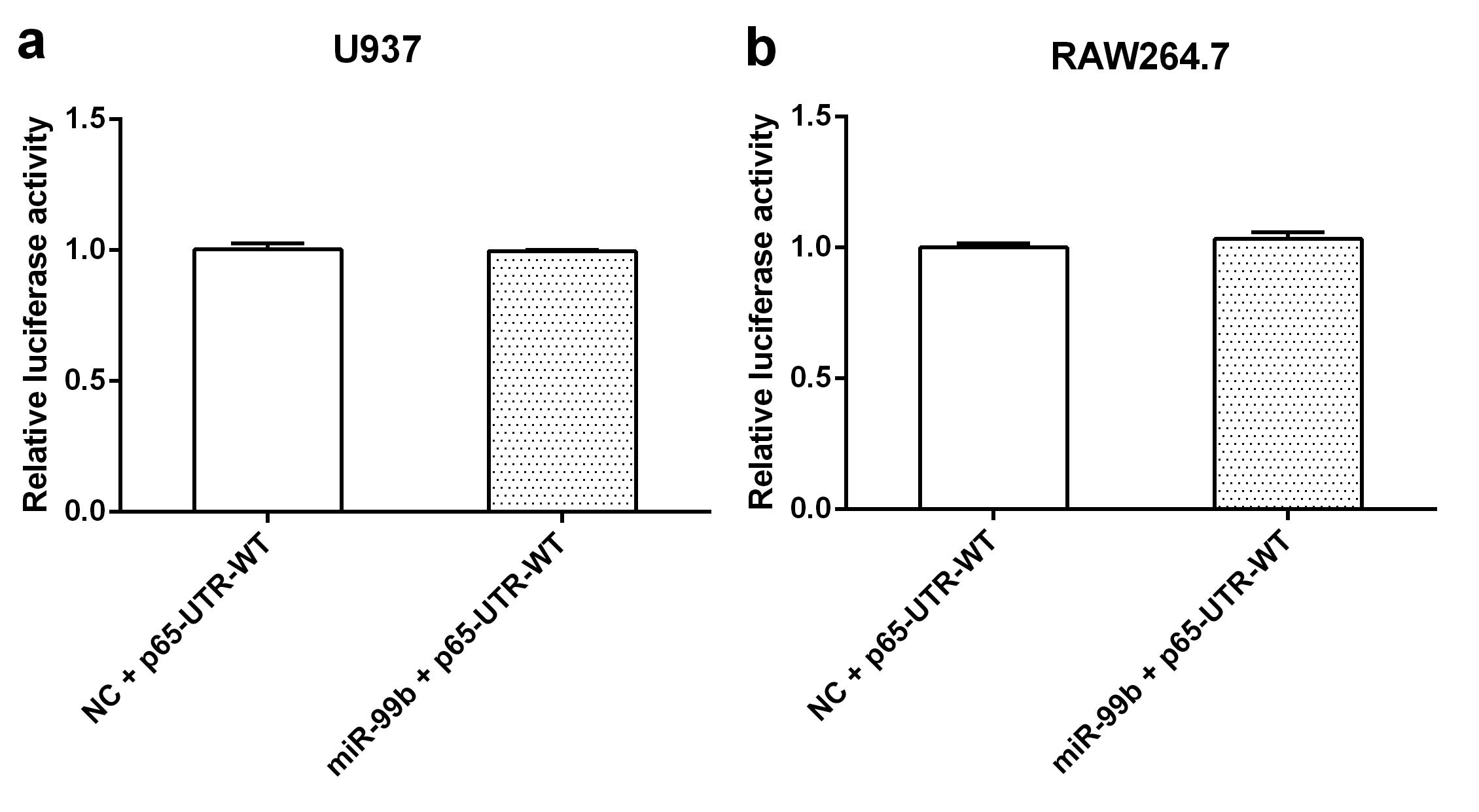

Supplement: Supplementary file 11 — S10 Figure [file 41426_2018_162_MOESM11_ESM.tif]

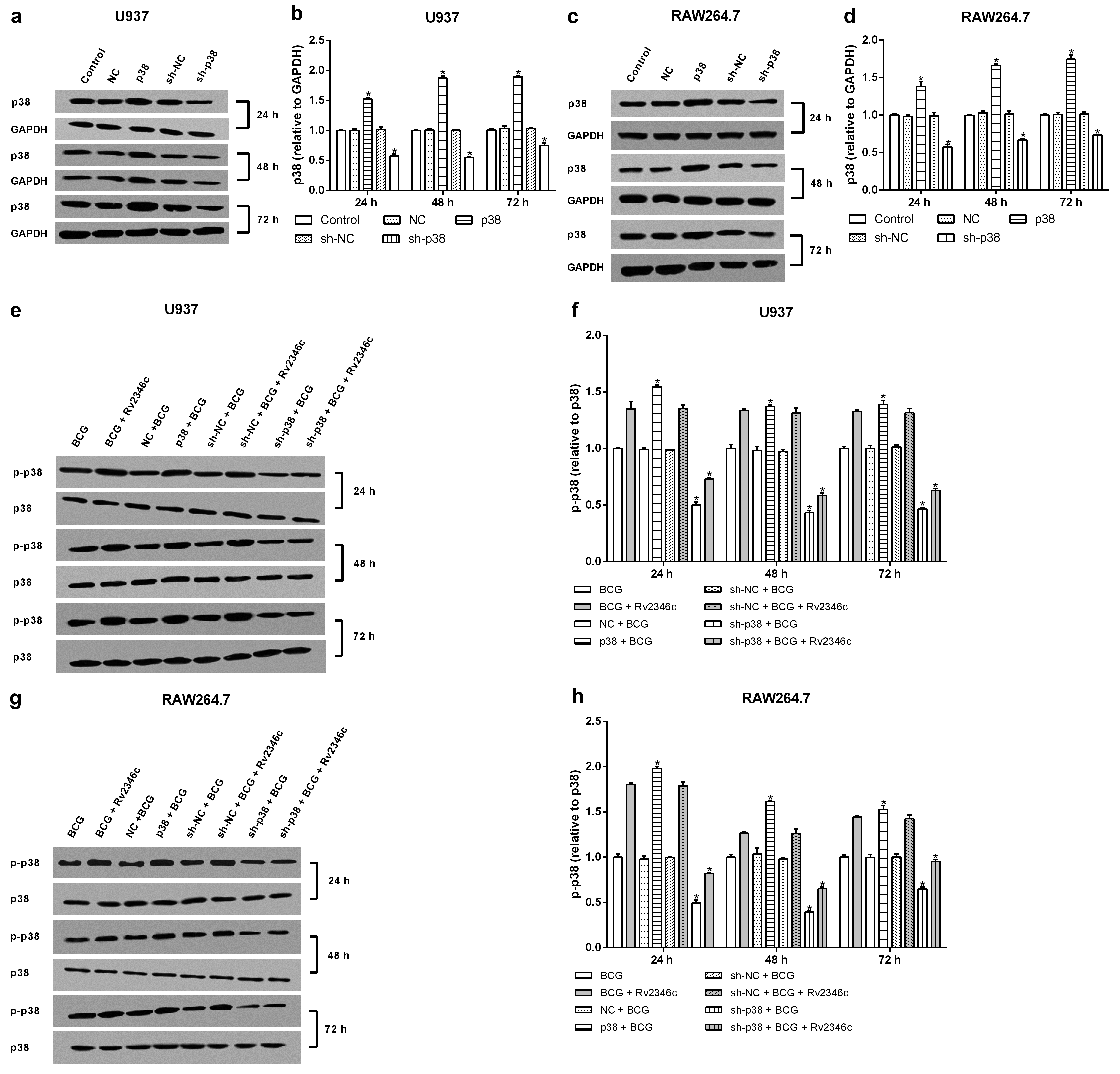

Supplement: Supplementary file 12 — S11 Figure [file 41426_2018_162_MOESM12_ESM.tif]
